# Neuropathology of Lewy body dementia: Lewy-related pathology, α-synuclein oligomers, and comorbid pathologies

**DOI:** 10.1186/s13024-025-00900-6

**Published:** 2025-11-03

**Authors:** Hiroaki Sekiya, Tomoyasu Matsubara, Michael A. DeTure, Dennis W. Dickson

**Affiliations:** 1 Department of Neuroscience, Mayo Clinic, 4500 San Pablo Road, Jacksonville, FL 32224 USA; 2 Department of Clinical Neuroscience and Therapeutics, Hiroshima University Graduate School of Biomedical and Health Sciences, Hiroshima, Japan

**Keywords:** Lewy body dementia, Dementia with Lewy bodies, Parkinson’s disease dementia, Alpha-synuclein, Oligomers, Comorbid pathology, Prion-like propagation, Machine learning algorithms, Neuropathology, Lewy bodies

## Abstract

Lewy body dementia is the second most common form of neurodegenerative dementia, following Alzheimer’s disease. This umbrella term encompasses dementia with Lewy bodies (DLB) and Parkinson’s disease dementia (PDD). The distinction between these two conditions lies in the timing of the onset of cognitive impairment relative to motor symptoms. In DLB, cognitive impairment precedes or coincides with motor symptoms within the first year, whereas in PDD, cognitive decline occurs more than a year after the onset of motor symptoms. Clinically, in addition to cognitive decline, patients with Lewy body dementia have parkinsonism, visual hallucinations, and fluctuations of cognitive status. The pathological hallmark of this condition is the presence of Lewy bodies and Lewy neurites, collectively referred to as Lewy-related pathology. This is identical to Parkinson’s disease, where dementia is not observed. The principal component of Lewy-related pathology is α-synuclein, which classifies this disorder as an α-synucleinopathy. While Lewy-related pathology represents a later stage of α-synuclein aggregation, earlier stages involve α-synuclein oligomers. Emerging evidence suggests α-synuclein oligomers may be more toxic than Lewy-related pathology. In addition to α-synuclein pathology, previous studies frequently observed comorbid pathological conditions, including Alzheimer’s disease neuropathologic change, TAR DNA-binding protein 43 (TDP-43) pathology, and cerebral small vessel disease among others. In this review, we provide a comprehensive overview of the underlying pathologies for Lewy body dementia and their molecular mechanisms and clinical implications. We also discuss concepts including the prion-like propagation hypothesis of α-synuclein, α-synuclein strain hypothesis, and recent advances in machine learning algorithms for analyzing propagation patterns. The purpose of this manuscript is to elucidate these complex pathological conditions, advance our understanding of the disease, and improve diagnostic strategies.

**Supplementary Information:**

The online version contains supplementary material available at 10.1186/s13024-025-00900-6.

## Introduction

Dementia with Lewy bodies (DLB) is the second most common form of neurodegenerative dementia after Alzheimer’s disease (AD). Clinical manifestations of this condition include recurrent visual hallucinations, fluctuations in cognitive status, REM sleep behavior disorder (RBD), and parkinsonism, in addition to cognitive decline [1]. Parkinson’s disease (PD) is a movement disorder that presents with extrapyramidal symptoms including bradykinesia, rigidity, and tremor. In addition to these motor symptoms, increasing attention has been focused on nonmotor symptoms, including cognitive dysfunction [2, 3]. Indeed, dementia prevalence increases over the course of PD, with more than 80% of individuals surviving 20 years developing dementia [4]. This condition is referred to as Parkinson’s disease dementia (PDD). “Lewy body dementia” is the collective term for DLB and PDD. The one-year rule distinguishes DLB and PDD; DLB is diagnosed when the patient presents with dementia before or within one year after the onset of parkinsonism, while PDD is diagnosed when the patient develops dementia more than one year after the onset of parkinsonism. If patients never develop dementia, the diagnosis is PD.

The pathological hallmark of Lewy body dementia is eosinophilic intraneuronal inclusions called Lewy bodies. Lewy bodies are observed in the cytoplasm of neurons and are mainly composed of pathological aggregates of α-synuclein [5, 6]. Morphologically, Lewy bodies include brainstem-type with a characteristic halo and cortical-type without a distinct halo. Pathological aggregates of α-synuclein are also detected in dystrophic neurites, called Lewy neurites. Collectively, Lewy bodies and Lewy neurites are referred to as Lewy-related pathology. While Lewy-related pathology represents a late-stage aggregate composed of α-synuclein fibrils, α-synuclein forms oligomers consisting of several molecules in the early phase of its aggregation, which may be in equilibrium with α-synuclein fibrils [7, 8]. Recently, there has been growing interest in the potential neurotoxicity of these early-stage α-synuclein aggregates [9–11]. Furthermore, the frequent co-occurrence of other pathological conditions in Lewy body dementia has been described, including the accumulation of non-α-synuclein protein aggregates and vascular pathology [12–14].

This review provides a comprehensive overview of the underlying neuropathology in Lewy body dementia, ranging from the conventional Lewy-related pathology to α-synuclein oligomers and comorbid pathologies, and suggests directions for future research.

## Terminology

In this field, inconsistent use of terms leads to confusion in the terminology [15]. In particular, the abbreviation “LBD” is used for both Lewy body disease and Lewy body dementia, further compounding this confusion. Therefore, we begin this review by reaffirming the definitions of each term. While the Diagnostic and Statistical Manual of Mental Disorders, Fifth Edition (DSM-5) has replaced “dementia” with “major neurocognitive disorder” [16], terms such as “Lewy body dementia,” “dementia with Lewy bodies,” and “Parkinson’s disease dementia” continue to be widely used in clinical practice and research. For consistency with current literature, we use these established terms throughout this review, while acknowledging the evolving nature of diagnostic terminology. Similarly, definitions of “cognitive impairment” and “dementia” may vary across studies cited in this review. We have generally preserved original terminology used in each research paper while acknowledging heterogeneity in diagnostic criteria and assessment methods. Therefore, the threshold distinguishing cognitive impairment from dementia may differ between studies due to methodological differences.

**Fig. 1:**
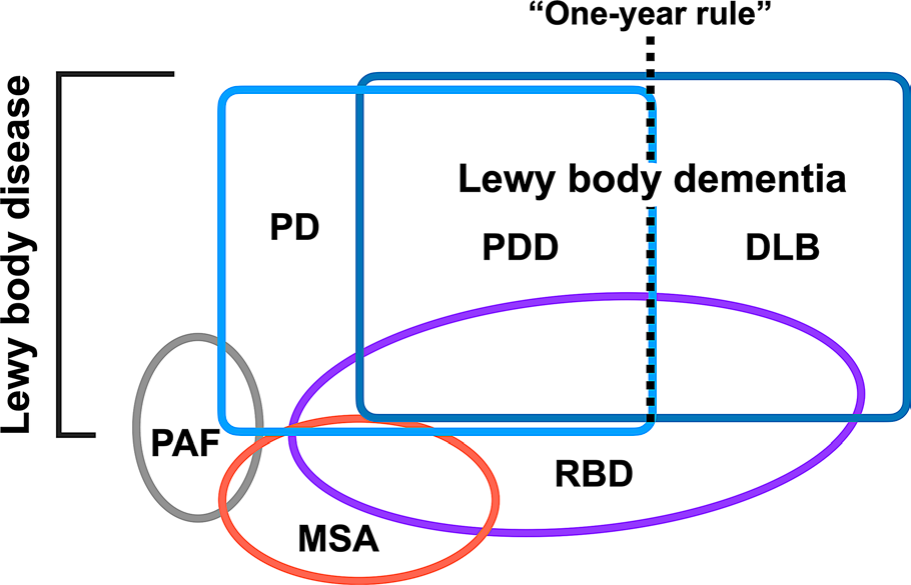
Terminology of α-synucleinopathy. DLB, dementia with Lewy bodies; MSA, multiple system atrophy; PAF, pure autonomic failure; PD, Parkinson’s disease; PDD, Parkinson’s disease dementia; RBD, REM sleep behavior disorder

Figure 1 illustrates the terminology associated with α-synucleinopathy. Pathologically, neurodegenerative disorders characterized by pathological aggregation of α-synuclein are collectively referred to as α-synucleinopathies [17]. Lewy body disease is a major category of α-synucleinopathies characterized by the presence of Lewy bodies, which are α-synuclein-positive spherical inclusions in the neuronal cytoplasm [5]. Lewy body disease encompasses DLB, PD, PDD, and pure autonomic failure. These disorders share the common pathological feature of Lewy bodies but differ in their clinical presentation and progression. Another significant α-synucleinopathy is multiple system atrophy (MSA), characterized by widespread and abundant glial cytoplasmic inclusions [18, 19]. Glial cytoplasmic inclusions are α-synuclein-positive triangular or oval inclusions found in the oligodendroglial cytoplasm [20]. Although neurons also contain α-synuclein aggregates [21–23], these glial cytoplasmic inclusions remain the pathological hallmark.

Patients with DLB present with cognitive impairment along with parkinsonism, visual hallucinations, RBD, and cognitive fluctuations. In contrast, patients with PD primarily exhibit extrapyramidal motor symptoms and a variety of non-motor symptoms. Although patients with PD do not initially present with cognitive impairment, a certain proportion develop dementia over time [24], and their condition is subsequently categorized as PDD. DLB and PDD are collectively referred to as Lewy body dementia.

It is important to note that while PD, PDD, DLB, and Lewy body dementia are clinical terms used for diagnosis based on clinical presentation, Lewy body disease is a pathological term. Lewy body disease refers to the presence of Lewy bodies in the brain, which can be definitively confirmed only through post-mortem examination. Lewy body disease is further classified into diffuse Lewy body disease, transitional Lewy body disease, and brainstem-predominant Lewy body disease based on the distribution of Lewy bodies. Generally, brainstem-predominant Lewy body disease corresponds to clinical PD, while transitional and diffuse Lewy body disease are more commonly associated with clinical DLB, although clinicopathological correlations can be complex.

In this review, while the abbreviations PD, PDD, and DLB will be used, the full spelling will be used for Lewy body disease and Lewy body dementia instead of using abbreviations to avoid confusion.

## Clinical and pathological spectrum of PDD and DLB

PDD and DLB are diagnosed based on the one-year rule in clinical practice as described above. The relationship between these two conditions has been debated; some researchers consider them as distinct entities and others view them as different manifestations of the same disease.

Table 1 summarizes the similarities and differences in clinical features between PDD and DLB. Many of the clinical differences between PDD and DLB naturally reflect their diagnostic definitions – DLB presents with early cognitive symptoms while motor symptoms are the initial manifestation in PDD. However, the pattern of disease progression appears to show more intrinsic differences beyond these definition-based distinctions. For instance, DLB patients tend to show more severe postural instability and gait difficulty despite having less severe parkinsonism overall. Additionally, they often experience more frequent falls early in the disease course compared to PDD patients. While both conditions eventually manifest similar symptoms, these differences in disease progression suggest potential differences in disease mechanisms.

**Table 1:** Similarities and differences in clinical features between PDD and DLB

Clinical symptoms	Similarities	Differences
Bradykinesia	Core feature of parkinsonism (slowness of movement and difficulty initiating movements)	Prevalence: cardinal symptom in nearly all PDD; present in up to 87% of DLB [25] Severity: Higher at baseline in PDD [26] Progression: Associated with more rapid decline in DLB [26]
Tremor	Can present as a resting tremor (rhythmic, occurs when the affected limb is at rest)	Type: Mixed pattern (resting and postural/action) can be seen in DLB [27] Prevalence: Common in PDD; variable in DLB (25–74%) [25, 27, 28] Progression: Lower risk of developing dementia in tremor-dominant PD [29, 30]
Rigidity	Common parkinsonian feature (increased muscle tone and resistance to passive movement)	Prevalence: Common in PDD; 48% in DLB [26]
Postural instability, recurrent falls	Implications for disease progression and patient mobility [31, 32]; Associated with higher risk of developing dementia in PD [33]	Prevalence: 48–73% in PDD [34, 35]; 35–40% in DLB [25, 34] Diagnostic value: Falls in 5 years preceding diagnosis more strongly associated with DLB [36]
RBD	Onset: Often precedes other symptoms [37–42] Impact: associated with more severe disease course [38, 41, 43, 44]	Prevalence: higher in DLB (50–90%) than PDD (likely similar to or slightly higher than PD; 25–58%) [39–41, 45]
Visual hallucinations	Complex, well-formed, unpleasant hallucinations [46] Associated with worse outcomes (more rapid cognitive decline, increased mortality, higher caregiver burden, increased risk of nursing home placement) [47, 48]	Prevalence: Higher in DLB vs PDD (72% vs 50% [49], 63% vs 20% [50], 51% vs 29% [51], 32% vs 11% [48]); 62% in DLB by meta-analysis in DLB [52]
Delusions	Associated with more rapid cognitive decline and increased caregiver burden [51]	Prevalence: Higher in DLB vs PDD (57% vs 29% [49], 52% vs 29% [51]) Severity: Often more pronounced in DLB [51]
Cognitive fluctuations	Severity: Comparable fluctuating attention [53]	Prevalence: Higher in DLB (25%) vs PDD (9%) [50]
Cognitive profiles	Comparable visuospatial abilities at baseline and progression [54]	More severe memory impairment in DLB and more severe executive impairment in PDD at baseline; Faster decline in executive function in PDD and faster decline in language ability in DLB [54] Worse inhibitory control and semantic fluency in DLB [55]
Orthostatic hypotension	Prevalence: 49% in PDD, 52% in DLB [56] Prognosis: Associated with shorter survival [57]	
Urinary incontinence	Associated with poor prognosis [57]	Prevalence: 50–97% in DLB [58–60]; in PDD, likely similar to that in PD (30% by meta-analysis [61])

Table 2 summarizes the pathological similarities and differences between PDD and DLB. Although findings are not always consistent across studies, DLB generally shows greater pathological burden compared to PDD in terms of α-synuclein, tau, amyloid β, and cerebral amyloid angiopathy. The discrepancies between studies may be attributed to variations in antibodies used and methods for evaluating pathological burden.

**Table 2:** Similarities and differences in pathological findings between PDD and DLB

References	Subjects	Brain regions	Methods	Findings
**Similarities**				
Harding et al. (2001) [62]	25 PD, 16 PDD, 24 DLB	Cortex (frontal, temporal, cingulate, and parahippocampal)	LB counts on α-synuclein IHC (LB509)	Comparable LB counts in cortex
Tsuboi et al. (2005) [63]	13 PD, 6 PDD, 28 DLB	Cortex (frontal, temporal, parietal, cingulate and parahippocampal), hippocampus, and amygdala	LB counts on NACP-IHC at x200 magnification, SP and NFT counts on thioflavin S	Comparable LB counts in cortex and amygdala, SP counts in cortex and hippocampus, and NFT counts in hippocampus
Tsuboi et al. (2007) [64]	7 PD, 7 PDD, 14 DLB	Putamen	% stained area of α-synuclein IHC	Comparable % stained area in putamen
Kalaitzakis et al. (2009) [65]	20 PD, 12 PDD, 7 DLB	Claustrum	Semi-quantitative assessment of Aβ (4G8) IHC	Comparable Aβ in claustrum
Kalaitzakis et al. (2011) [66]	52 PD, 41 PDD, 14 DLB, 7 MSA, 12 PSP	Putamen and caudate nucleus	Semi-quantitative assessment of Aβ (4G8) IHC	Comparable Aβ in putamen and caudate nucleus
Sierra et al. (2016) [67]	10 PD, 10 PDD, 10 DLB, 10 AD, 10 Ctrl	Midbrain and Cerebellum	Semi-quantitative assessment of α-synuclein (KM51), p-tau (AT8), and Aβ (6F/3D) IHC	Similar α-synuclein, Aβ, and p-tau burden in midbrain and comparable Aβ burden in cerebellum
**Differences**				
Jellinger et al. (2006) [68]	17 PDD, 17 DLB	Striatum	Semi-quantitative assessment of LRP, p-tau (AT8), and Aβ (4G8) IHC	A greater burden of LRP, Aβ plaques and tau pathology in striatum in DLB
Kalaitzakis et al. (2009) [65]	20 PD, 12 PDD, 7 DLB	Claustrum	Semi-quantitative assessment of α-synuclein (BD) IHC	A greater α-synuclein burden in claustrum in DLB
Halliday et al. (2011) [69]	7 PD, 12 PDD, 10 DLB, 5 ctrl	Striatum	Semi-quantitative assessment of Aβ (IE8) IHC	A greater burden of striatal Aβ plaques in DLB
Ruffmann et al. (2015) [70]	49 PD, 55 PDD, 17 DLB	Cortex, striatum	Semi-quantitative assessment of α-synuclein (42 or 211) and Aβ (4G8) IHC	A greater α-synuclein burden in temporal and parietal cortices and greater Aβ burden in striatum and frontal cortex in DLB
Hepp et al. (2016) [71]	27 PD, 56 PDD, 50 DLB	Cortex, amygdala, striatum	% stained area of Aβ (6F/3D) IHC	A greater Aβ burden in frontal, temporal, occipital, entorhinal cortices and caudate nucleus in DLB
Jellinger (2021) [72]	60 PD, 110 PDD, 60 DLB	Cortex	Semi-quantitative assessment of CAA severity with Aβ (4G8) IHC	More severe CAA in DLB
Jellinger (2023) [73]	100 PD, 110 PDD, 80 DLB	Cortex	Semi-quantitative assessment of CAA severity with Aβ (4G8) IHC	More severe CAA in DLB

Aβ, amyloid β; AD, Alzheimer’s disease; Ctrl, control: DLB, dementia with Lewy bodies; IHC, immunohistochemistry; LB, Lewy body; LRP, Lewy-related pathology; MSA, multiple system atrophy; NFT, neurofibrillary tangle; PD, Parkinson’s disease; PDD, Parkinson’s disease dementia; PSP, progressive supranuclear palsy; p-tau, phosphorylated tau; SP, senile plaque

Although the one-year rule is somewhat arbitrary, it is useful and widely used as a classification in clinical practice and in research. This distinction helps in the differential diagnosis and management of PDD and DLB, which, despite their overlap, may have different therapeutic and prognostic implications. For example, DLB often presents with higher amyloid and tau pathology compared to PDD [74]. In research, maintaining this distinction aids in the stratification of study populations, ensuring more homogeneous groups for clinical trials and epidemiological studies. This can improve the validity and reliability of research findings and facilitate the development of targeted therapies [75].

## Epidemiology

DLB is now recognized as one of the most common causes of dementia, affecting up to 5% of the general population and accounting for up to 31% of patients with dementia. The incidence of DLB is estimated to be 0.5–1.6 per 1,000 person-years in the general population and 32–38 per 1,000 person-years in patients with dementia [76–78]. Regarding PDD, a systematic review revealed that PDD accounts for 25–31% of patients with PD and represents 3.2–5.3% of all patients with dementia [24].

The wide ranges observed may be attributed to differences in diagnostic criteria, study methodologies, and population characteristics across studies. It should be noted that not all epidemiological studies differentiate PDD and DLB. Furthermore, the accurate diagnosis of Lewy body dementia in clinical practice remains challenging [79, 80].

A study of clinicopathologic correlations in 273 consecutive autopsies of nursing home residents reported that Lewy bodies were found in 17% [81]. In a combination of two cohorts, the Rush Religious Order Study and the Memory and Aging Project, 18% of 872 autopsies had Lewy bodies [82]. A prospective cohort study from Hisayama town in Japan showed that among 1,266 autopsies (with an approximate autopsy rate of 75% in the town), the prevalence of DLB was 4.4% in the general population and 14.4% in patients with dementia, respectively [83].

The prevalence of dementia in PD increases over the disease course, with approximately 80% of PD patients developing dementia (i.e., PDD) after 20 years from motor symptom onset [4]. Given the increasing prevalence of PD [84], the prevalence of PDD is expected to rise in the future.

## Genetics of Lewy body dementia

Lewy body dementia is characterized by several genetic features, which have been identified through various genetic association studies. The most consistently replicated genetic associations involve mutations in the *APOE*, *GBA1*, and *SNCA* genes [85].

The *APOE* gene encodes apolipoprotein E and is polymorphic with three major alleles: ε2, ε3, and ε4 [86, 87]. The *APOE* ε4 allele is a significant genetic risk factor for Lewy body dementia and influences the disease’s progression and Lewy-related pathology [88–91]. Although the *APOE* ε4 is the strongest risk factor for AD [92–95], a study of 652 autopsy-confirmed Lewy body disease demonstrated that patients with *APOE* ε4 had higher Lewy body burden in the cortices, independent of AD pathology [96]. Furthermore, in a study involving 81 PDD, 91 DLB without AD pathology, 224 Lewy body disease with AD pathology, 244 AD, and 269 control cases, the frequency of *APOE* ε4 was significantly higher in PDD and DLB without AD pathology compared to controls [97]. While many studies have shown that *APOE* ε4 is associated with Lewy body dementia, it should be noted that a recent study did not confirm the involvement of *APOE* ε4 in DLB without AD pathology [98]. Clinically, a study of 3,923 patients with PD showed that 6.7% converted to PDD during the observation period, and that *APOE* ε4 was associated with the development of dementia [99]. Several studies have also reported that *APOE* ε4 carriers with PD or Lewy body dementia experience more rapid cognitive decline [100–103]. Experimental studies using mouse models also suggest the association between APOE ε4 and α-synuclein pathology. One study showed that *APOE* ε4 increased α-synuclein pathology and impaired behavioral performance independently of amyloid pathology [104]. In a study using *SNCA* A53T transgenic mice, mice on *APOE* ε4 showed more abundant phosphorylated α-synuclein pathology in the brainstem compared to *APOE* knockout, ε2, ε3 mice, and had more α-synuclein accumulation in the substantia nigra pars compacta than ε2 or *APOE* knockout mice [105].

The *GBA1* gene encodes the lysosomal enzyme, glucocerebrosidase. While homozygous mutations in the *GBA1* gene cause Gaucher disease, heterozygous mutations in the *GBA1* gene are a significant risk factor for PD and Lewy body dementia [106]. Many studies have demonstrated the association between *GBA1* gene variants and Lewy body dementia [90, 107–111]. Reduced glucocerebrosidase levels contribute to increased α-synuclein accumulation in Lewy body dementia, partly through elevating glucosylsphingosine levels, ultimately impacting disease manifestation [112]. Clinical characteristics of Lewy body dementia with *GBA1* mutations include younger onset, more frequent and severe parkinsonism, and higher frequency of visual hallucinations and fluctuations [113]. Liu et al. screened 1,921 patients with PD for *GBA1* mutations and found that carriers of neuropathic Gaucher disease mutations and complex *GBA1* alleles exhibited a more rapid decline in Mini-Mental State Examination (MMSE) scores, whereas this association was not observed in carriers of non-neuropathic mutations or risk variants [114]. Mata et al. analyzed 1,369 patients with PD for *GBA1 mutations* and reported that both *GBA1* mutations and the E326K polymorphism were associated with poorer performance in working memory/executive function and visuospatial abilities [115]. Pathologically, one study revealed an inverse relationship between the frequency of *GBA1* mutations/polymorphisms and the severity of comorbid AD in Lewy body dementia [116], suggesting that *GBA1* genetic variants may influence the development of Lewy body dementia independent of comorbid AD. A recent study using 943 autopsy-confirmed Lewy body disease found that those with *GBA1* mutations exhibited lower Braak neurofibrillary tangle stage and Thal amyloid phase and more severe Lewy-related pathology [117].

The *SNCA* gene, which encodes α-synuclein, is another critical genetic factor in Lewy body dementia [85, 88]. A point mutation in the *SNCA* gene (A53T) was first reported in a large pedigree of familial PD [118, 119]. Since then, other point mutations including V15A, A30P/G, E46K, H50Q, and G51D, A53E/V, T72M, E83Q, as well as duplications, and triplications have been described [120–136]. The pathologic findings of SNCA point mutations based on postmortem human brain samples are summarized in Table 3. Many point mutations show more severe pathology compared to sporadic PD, and some mutations have been reported to exhibit glial cytoplasmic inclusions. Of note, these point mutations in SNCA have been reported to promote oligomer formation of α-synuclein (α-synuclein oligomers will be discussed in more detail later) [142–145]. A study with 198 PDD and 922 DLB patients identified novel *SNCA* gene variations associated with PDD (rs10018362, rs7689942) and DLB (rs974711, rs1348224), with rs1348224 showing association in both conditions, suggesting both shared and distinct genetic risk factors within *SNCA* for these disorders [146]. A meta-analysis study using data from 59 families with SNCA multiplications revealed that SNCA multiplications were associated with the development of Lewy body dementia and that higher SNCA copy numbers correlated with earlier onset of cognitive impairment [147]. As for cognitive function, a recent study compared 16 patients with PD carrying the SNCA A53T mutation to age- and disease duration-matched idiopathic PD patients. While Montreal Cognitive Assessment score was comparable at baseline, the A53T group demonstrated greater cognitive decline over the three-year follow-up period. Moreover, executive and visuospatial functions were poorer in the A53T group at baseline compared to idiopathic PD and further deteriorated during disease progression [148].

**Table 3:** Pathological features of patients with SNCA point mutations

Mutations	References	AAD	Sex	Duration	Description
A30P	Seidel et al. (2010) [126]	69	M	15	Neuronal loss in SNpc, LC, dmX Widespread LRP Glial inclusions
E46K	Zarranz et al. (2004) [124]	69	M	8	Neuronal loss in dmX and SNpc Widespread LRP Microspongiosis in SNpc
A53E	Pasanen et al. (2014) [131]	60	F	24	Neuronal loss in SNpc, hippocampus, cortices (temporal, insular, cingulate) A few LBs in dmX Neuronal and glial cytoplasmic inclusions in putamen, caudatus, amygdala, hippocampus, cortices (temporal, insular, cingulate)
A53T	Golbe et al. (1990) [118] and Duda et al. (2002) [137]	49	M	11	Neuronal loss in SNpc, hippocampus LBs in SNpc, LC, dmX, raphe nucleus, nbM, limbic cortices LNs in LC, dmX, amygdala, CA2, CA3, parahippocampal gyrus, striatum, motor cortex
A53T	Golbe et al. (1990) [118]	64	M	4	Neuronal loss in LC LBs in LC *Only cerebral cortex, basal ganglia, pons, cerebellum, and cervical cord available
A53T	Spira et al. (2001) [138]	47	M	5	Neuronal loss in SNpc, LC, CA2, CA3 LBs in SNpc and LC LNs in SNpc, LC, dmX, CA2, CA3, cortices Vacuolation in temporal cortex
A53T	Spira et al. (2001) [138]	55	M	9	Neuronal loss in SNpc, LC, CA2, CA3, putamen, globus pallidus, temporal cortex LBs in SNpc, LC, oculomotor nucleus LNs in SNpc, LC, dmX, oculomotor nucleus No glial inclusions
A53T	Yamaguchiet al. (2005) [139]	50	F	9	Neuronal loss in dmX, SNpc, nbM, limbic, cortex LBs in LC, amygdala, hippocampus, temporal cortex; more widespread and abundant LNs Microvacuolation in nbM, nucleus accumbens, cortices (entorhinal, cingulate, temporal)
A53T	Markopoulou et al. (2008) [140]	71	F	12	Neuronal loss in SNpc, LC, nbM, amygdala, CA1, subiculum, temporal cortex LRP in dmX, LC, SNpc, nbM, hypothalamus, limbic cortices
A53T	Markopoulou et al. (2008) [140]	39	M	7	Neuronal loss in SNpc, nbM, CA2 LRP in dmX, LC, SNpc, nbM, amygdala, limbic cortices Glial cytoplasmic inclusions in limbic and temporal cortices
A53T	Nishioka et al. (2020) [141]	52	M	10	Neuronal loss in SNpc, LC, dmX Widespread Lewy-related pathology LB Mild glial cytoplasmic inclusions in amygdala, hippocampus, cortices (frontal, temporal, parietal, occipital)
H50Q	Proukakis et al. (2013) [129] and Kiely et al. (2015) [130]	83	F	12	Neuronal loss in SNpc LRP in SNpc, putamen, caudate nucleus, cortex (Braak PD stage 6) No glial inclusions
G51D	Kiely et al. (2013) [127] and Kiely et al. (2015) [130]	48	M	19	Neuronal loss in SNpc, LC, dmX, CA2, CA3, cortices (temporal and insular) Various morphology of neuronal α-synuclein cytoplasmic inclusions in wide regions including cortices Glial cytoplasmic inclusions
G51D	Lesage et al. (2013) [127]	67	F	7	Neuronal loss in SNpc, LC, putamen, caudate nucleus, globus pallidus, thalamus LBs in SNpc, LC, dmX, hippocampus, cingulate cortex LNs in spinal cord, SNpc, LC, putamen, caudate nucleus, globus pallidus, hippocampus, cortices (frontal, cingulate, motor) Annular or crescent neuronal cytoplasmic inclusions in spinal cord, LC, dmX, hippocampus, cortices (cingulate, motor)
G51D	Kiely et al. (2015) [130]	75	ND	6	Neuronal loss in SNpc, LC, dmX, CA2, CA3, cingulate cortex Various morphology of neuronal α-synuclein cytoplasmic inclusions in wide regions including cortices Glial cytoplasmic inclusions
G51D	Kiely et al. (2015) [130]	52	ND	6	Neuronal loss in SNpc, LC, dmX Various morphology of neuronal α-synuclein cytoplasmic inclusions in wide regions including cortices Glial cytoplasmic inclusions
E83Q	Kapasi et al. (2020) [133]	63	F	4	Neuronal loss in SNpc, amygdala, cortices (entorhinal, cingulate, temporal) LBs in SNpc, amygdala, basal ganglia, thalamus, CA1, cortices (entorhinal, cingulate, temporal, frontal, parietal) Spongiform changes in cortices (entorhinal, cingulate, temporal)

No autopsy report was found for patients with SNCA V15A, A30G, A53V, or T72M

*AAD*, age at death; *dmX*, dorsal motor nucleus of the vagus; *F*, female; *LBs*, Lewy bodies; *LC*, locus coeruleus; *LNs*, Lewy neurites; *LRP*, Lewy-related pathology; *M*, male; *nbM*, nucleus basalis of Meynert; *ND*, no data; *SNpc*, substantia nigra parc compacta

Furthermore, *SNCB* has also been linked to Lewy body dementia. β-synuclein, encoded by *SNCB*, inhibits α-synuclein aggregation [149] and α-synuclein oligomer formation [150], and cortical β-synuclein levels are reduced in diffuse Lewy body disease without comorbid AD pathology [151]. Specific insertion and deletion mutations in the *SNCB* gene are frequently observed in DLB, potentially contributing to the decrease of β-synuclein [152]. Conversely, β-synuclein accumulation is observed in dopaminergic neurons [153], and mutations such as V70M and P123H in *SNCB* [154] are associated with DLB and exacerbate neurodegeneration [155].

Additionally, correlations between MAPT haplotypes and Lewy body dementia have been documented. The MAPT H1/H1 haplotype is associated with more severe synuclein burden [156]. While the MAPT H2 haplotype has a protective effect, a relatively rare H1G haplotype is linked to an increased risk of DLB [157]. The rare H1j haplotype significantly correlates with neurodegeneration in the putamen [158].

## Lewy-related pathology

The pathological hallmark of PD and DLB is Lewy bodies and Lewy neurites, which are collectively referred to as Lewy-related pathology [159–163]. Lewy bodies are round inclusions in cytoplasm of neurons, while Lewy neurites are aggregations in the dystrophic neurites. Lewy bodies are ultrastructurally assemblies of filamentous and amorphous granular material [164], the major component of which is α-synuclein consisting of 140 amino acid residues [5], with phosphorylation at serine 129 [165]. While fibrillar α-synuclein forms the structural backbone of Lewy bodies, studies with immunohistochemistry [166–170] and proteomic analyses of isolated Lewy bodies [171, 172] have consistently demonstrated that ubiquitin, p62/SQSTM1, and phosphorylated neurofilament subunits also constitute Lewy bodies, reflecting activation of protein quality control pathways and cytoskeletal pathways. Furthermore, these proteomic analyses have catalogued over 100 additional proteins associated with Lewy bodies, highlighting that Lewy bodies are complex protein assemblies whose complete functional hierarchy remains to be elucidated.

### Macroscopic pathology

**Fig. 2:**
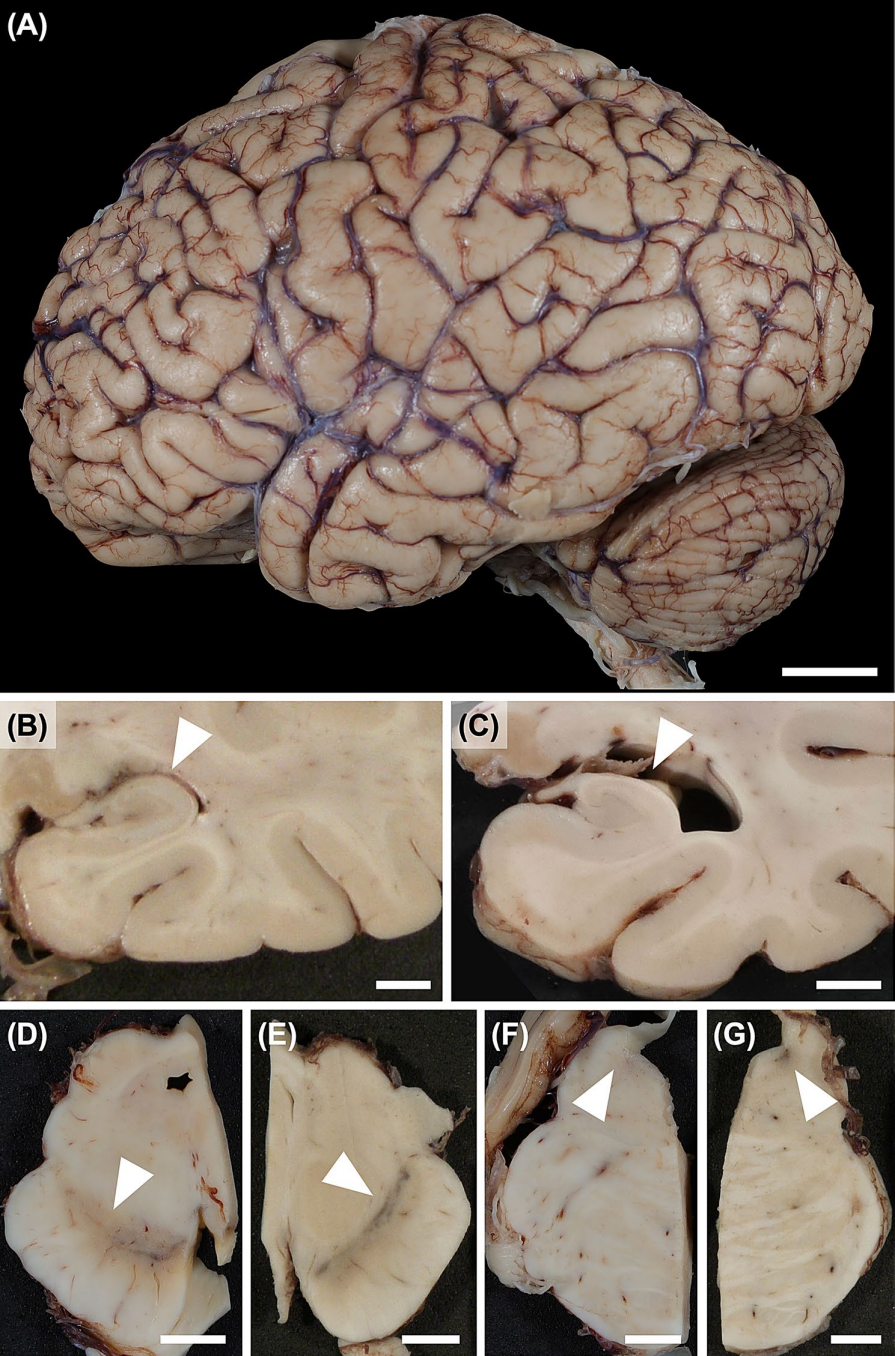
Macroscopic images. Lateral view of the brain with dementia with Lewy bodies shows no significant atrophy (**A**). Coronal brain sections at the level of the lateral geniculate body showing the hippocampus: Lewy body dementia without Alzheimer’s disease neuropathological changes (**B**) and Lewy body dementia with Alzheimer’s disease neuropathological changes (**C**). Substantia nigra and locus coeruleus in dementia with Lewy bodies shows loss of neuromelanin pigment (**D, F**) in contrast with a normal subject (**E, G**). Scale bars: 2 cm (**A**) and 0.5 cm (**B-G**)

In Lewy body dementia, the brain typically does not exhibit significant atrophy (Fig. 2A), and the brain weight remains within normal limits or only slightly lower than normal. If atrophy or a marked decrease in brain weight is observed, other diagnoses or the influence of co-pathologies such as comorbid AD should be considered (Fig. 2B, C). In the brainstem, Lewy body dementia shares the characteristic feature of depigmentation of both the substantia nigra and the locus coeruleus with PD (Fig. 2D, E). The substantia nigra is particularly susceptible in the ventrolateral and caudal segments [173]. In addition, atrophy or discoloration of the basal ganglia is usually not observed in Lewy body dementia.

### Microscopic pathology

**Fig. 3:**
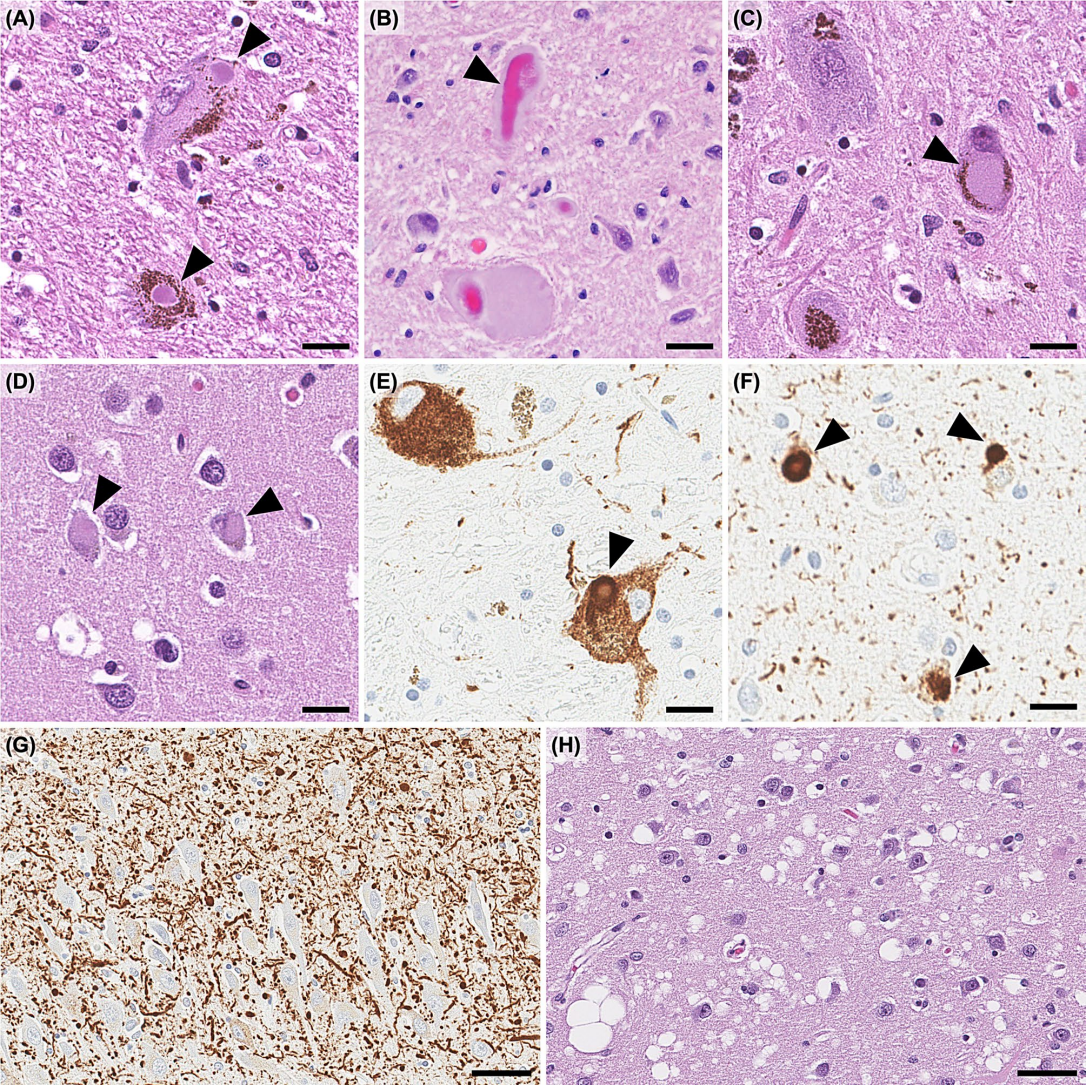
Microscopic images of Lewy body dementia. (**A**) brainstem Lewy bodies in the substantia nigra; (**B**) intraneuritic Lewy body in the dorsal motor nucleus of the vagal nerve; (**C**) pale body in the substantia nigra; (**D**) cortical Lewy bodies in the cingulate cortex; (**E**) brainstem Lewy body in the substantia nigra; (**F**) cortical Lewy bodies in the cingulate gyrus; (**G**) numerous Lewy neurites in the CA2 subfield of the hippocampus; (**H**) spongiform change in the pre-α layer of the entorhinal cortex. (**A-D, H**): hematoxylin and eosin staining, (**E-G**): immunohistochemistry for phosphorylated α-synuclein. Scale bars: 20 µm (**A-F**), 50 µm (**G, H**)

The histological hallmark of Lewy body dementia is Lewy bodies [174]. The classic Lewy body, mainly observed in the brainstem, is an intraneuronal cytoplasmic inclusion consisting of a dense and concentric hyaline eosinophilic core surrounded by a halo (Fig. 3A). Similar inclusions are also observed within neuronal processes, particularly in the dorsal motor nucleus of the vagal nerve and the peripheral sympathetic nerve, and are termed intraneuritic Lewy bodies [174, 175] (Fig. 3B). Less dense, mildly eosinophilic inclusions without a halo are referred to as pale bodies, often observed in the pigmented neurons of the substantia nigra, and are considered an immature form of Lewy bodies [176] (Fig. 3C). In the cortex, smaller and round intraneuronal eosinophilic cytoplasmic inclusions without a distinct halo are observed and termed cortical Lewy bodies [177] (Fig. 3D). Lewy bodies observed in the brainstem are typically relatively large, spherical, and strongly eosinophilic, characterized by a dense central core and a surrounding halo. In contrast, cortical Lewy bodies are relatively smaller, less intensely eosinophilic, and usually lack a halo. Despite these morphological differences, both types of Lewy bodies can be visualized using immunohistochemistry for α-synuclein (Fig. 3E, F) and ubiquitin [166]. Immunostaining for α-synuclein also allows the detection of Lewy neurites, which are not visible with hematoxylin and eosin staining [178] (Fig. 3G). Lewy neurites are generally observed more abundant than Lewy bodies in pathological sections. This is primarily due to their structural differences: Lewy neurites represent α-synuclein accumulation within long processes, whereas Lewy bodies are inclusions within the neuronal soma. Given that histological sections are typically thin (5–10 µm), elongated processes are more likely to be captured in any given plane of sectioning, while spherical soma may be missed depending on the cutting angle. Additionaly, although Lewy neurites appear more abundant than Lewy bodies, the burden of both pathologies tends to increase in nearly parallel as the disease progresses [179, 180]. Therefore, Lewy neurites and Lewy bodies are considered fundamentally the same pathology differing only in morphology. Immunohistochemistry has uncovered that Lewy bodies are widely distributed in both the central and peripheral nervous systems [181–184]. Microvacuolation of the neuropil is another characteristic of Lewy body dementia [185, 186], which is anatomically confined mainly to the limbic cortices, especially the amygdala and entorhinal cortex (Fig. 3H). Ultrastructural studies using electron microscopy have shown that these vacuoles are located within dendrites and/or axons, suggesting that they reflect degeneration of these neuronal processes [185, 187, 188]. This change occurs independently of any prion disease-related mechanisms, as immunohistochemistry with antibodies against prion protein failed to label plaques or vascular amyloid deposits [185]. The pattern and distribution of spongiform change in Lewy body disease also differ from those in prion diseases such as Creutzfeldt-Jakob disease, where spongiform change is widespread, confluent, and a core diagnostic criterion [189].

### Distribution of Lewy-related pathology

Lewy body disease can be classified based on the distribution pattern of Lewy bodies [1, 190] (Table 4). Kosaka et al. classified Lewy body disease into several categories: diffuse, transitional, and brainstem-predominant [190]. Diffuse Lewy body disease is the pathological diagnostic term for cases in which Lewy bodies are widely distributed in the neocortex, while brainstem-predominant Lewy body disease is used when Lewy bodies are mainly confined to the brainstem with minimal distribution in the cerebrum. Transitional Lewy body disease is used for cases in which the distribution of Lewy bodies is intermediate between the two, and the Lewy bodies are found in the limbic system, which is also referred to as limbic Lewy body disease. Braak et al. classified PD into 6 stages based on the extent of Lewy body distribution, ranging from minimally affected brains to those with widespread involvement [181] (Table 4). A group of patients was reported with amygdala-predominant Lewy bodies without notable Lewy bodies in the brainstem [192, 193]. These subtypes are often observed as a concomitant alteration in AD [193]. Moreover, AD with Lewy bodies often exhibits the coexistence of tau and α-synuclein in the same neuron [194–196] (comorbid AD will be discussed in detail below). While various staging schemes have been proposed [179, 183, 191, 197–199], the current pathological classification of Lewy body disease into five types is now widely used: diffuse (neocortical), transitional (limbic), brainstem-predominant, amygdala-predominant, and olfactory bulb-only [1]. In diffuse Lewy body disease, brainstem Lewy-related pathology is present in nearly all patients, to some extent. Even in individuals without clinically overt parkinsonism, Lewy-related pathology and neuronal loss in the substantia nigra pars compacta are often observed. This discrepancy can be explained by the “threshold effect”, which suggests that approximately 50–90% loss of nigral dopaminergic (tyrosine hydroxylase-positive) neurons is required for the manifestation of motor symptoms [200]. The dissociation between pathological changes and clinical symptoms in DLB may be attributed to neuronal loss below this threshold, precedence of cognitive symptoms, or different progression patterns of nigral pathology.

**Table 4:** Staging of Lewy body disease

Kosaka stage [190]	Braak PD stage [181]	Unified staging system [191]		Distribution of Lewy bodies
-	1	Olfactory bulb only (group I)		Olfactory bulb
Brainstem-predominant type (group C)		Brainstem-predominant type (group IIa)	Brainstem and limbic type (group III)	Medullary tegmentum and the dorsal motor nucleus of the vagal nerve
	2			Locus coeruleus, caudal raphe and reticular nucleus of medullary and pontine tegmentum
	3			Substantia nigra and basal forebrain
Transitional (limbic) type (group B)	4	Limbic predominant type (group IIb)		Medial temporal (limbic) cortices, including amygdala and CA2 region of the hippocampus
Diffuse cortical type (group A)	5	Neocortical type (group IV)		Multimodal association cortices, especially frontal and temporal lobes
	6			Unimodal association cortices and primary cortices

*PD*, Parkinson’s disease; CA, cornu ammonis

In early or incidental Lewy body disease, the distribution of Lewy bodies is often confined to the brainstem and peripheral autonomic nerves or to the olfactory bulb and amygdala [201, 202]; in advanced Lewy body disease, the distribution of Lewy bodies is more widespread throughout the brain, giving rise to the “propagation hypothesis” discussed below.

In addition to the distribution of Lewy-related pathology in the central nervous system, Lewy-related pathology is also observed in the peripheral nervous system, particularly in areas closely associated with autonomic function, such as the nerve plexus in the walls of the esophagus and intestines [182, 183, 203–205], submandibular gland [206, 207], nerve fibers in the skin [208–210], paraspinal sympathetic ganglia and sympathetic nerves surrounding the heart [184, 203, 211, 212], adrenal glands [213, 214], kidney and genitourinary tract [182, 215], and retina [216].

### Molecular mechanisms of neurodegeneration

Multiple molecular mechanisms are proposed to underlie neurodegeneration associated with Lewy-related pathology. First, loss of normal α-synuclein function results in decreased synaptic plasticity [217, 218], impaired neurotransmitter release [219, 220], abnormal SNARE complex formation [221, 222], vesicular transport defects [223, 224], and disrupted dopamine synthesis and transport [225–227]. Second, toxic gain-of-function mechanisms from abnormal α-synuclein aggregate accumulation cause axonal transport impairment [228, 229] and inhibition of protein degradation systems, including the lysosomal-autophagy [230, 231] and ubiquitin-proteasome pathways [232, 233]. Additionally, direct damage to intracellular organelles manifests as mitochondrial dysfunction [234–236], lysosomal membrane rupture [237, 238], and cytoskeletal disruption [239, 240]. Furthermore, prion-like mechanisms of intercellular transmission and aggregate spreading via extracellular vesicles contribute to pathological progression (pathology propagation will be discussed in detail later) [241, 242]. These complex mechanisms might interact synergistically, ultimately leading to neuronal death.

However, the relationship between Lewy-related pathology and neurodegeneration may be more complex than previously thought. Emerging evidence suggests that α-synuclein oligomers, which are though to precede Lewy-related pathology formation [243], exhibit greater toxicity than mature aggregates (α-synuclein oligomers will be discussed later) [9, 244]. This has led to the “protective aggregate” hypothesis, proposing that Lewy-related pathology formation may represent a cellular defense mechanism that sequesters highly toxic α-synuclein oligomers [245–247]. Supporting this concept, a *Drosophila* PD model demonstrated that inhibiting α-synuclein phosphorylation at serine 129 increased Lewy body-like inclusions while paradoxically suppressing dopamine neuron loss, whereas mimicking phosphorylation decreased inclusions but enhanced neurotoxicity [248]. Dynamic modeling and in silico studies have demonstrated that early anti-oligomer intervention provides neuroprotective effects while late-stage inhibition of aggregate formation proves counterproductive, and that rapid fibril formation from α-synuclein oligomers minimizes neuronal toxicity [249, 250]. These findings suggest a potential neuroprotective function of aggregate formation, although further investigation is needed to fully understand this complex relationship.

## Clinical implication of Lewy-related pathology

A large number of clinicopathological studies have been conducted on the impact of Lewy-related pathology on clinical manifestations. In one study using a prospectively followed cohort with 49 DLB patients, among α-synuclein, tau, and amyloid β, α-synuclein contributed most to reduced disease duration [251]. Another study also demonstrated that diffuse Lewy body disease had shorter disease duration compared to transitional Lewy body disease [252].

Regarding the overall distribution of Lewy-related pathology and clinical symptoms, studies have examined the correlation between pathological distribution based on the Unified Staging System for Lewy Body disorders and symptoms [253]. Among 280 patients, 9% were classified as stage 1 (olfactory bulb only), 15% as stage IIa (brainstem predominant), 14% as stage IIb (limbic predominant), 32% as stage III (brainstem and limbic), and 31% as stage IV (neocortical). Correlations between these stages and parkinsonism, cognitive impairment, RBD, and olfactory dysfunction have been reported.

### Cognitive impairment

The association between dementia and Lewy-related pathology has been investigated by examining differences in the degree of Lewy-related pathology between PD and PDD or DLB. An autopsy study including 20 PD and 22 PDD patients revealed that the severity of neocortical Lewy-related pathology was higher in PDD [254]. Another study with 25 PD, 16 PDD, and 24 DLB patients reported that the amount of Lewy-related pathology in the parahippocampal gyrus was most useful in differentiating PD from PDD or DLB [62]. Comparison between 9 PD and 12 PDD patients demonstrated that the number of Lewy bodies was much higher in the amygdala, limbic cortex, and neocortex in PDD [255]. In a study with 60 PD and 69 PDD patients, cortical Lewy body scores were significantly higher in PDD [256]. Furthermore, the severity of cortical Lewy-related pathology was higher in PDD in a study with 48 PD and 92 PDD patients [257], and a higher cortical Lewy body score contributed most to dementia in a study involving 46 PD and 109 PDD patients [258]. Regarding the distribution of Lewy-related pathology, PDD has a higher proportion of transitional or diffuse patterns compared to PD without dementia [255, 258].

In contrast, there are several reports indicating that cortical Lewy-related pathology is not necessarily associated with dementia. For example, 19% of 16 PDD patients or 24% of 34 PDD patients exhibited Lewy-related pathology confined to the brainstem [259, 260]. Another study examined the distribution pattern of Lewy-related pathology in 17 PD patients and 21 PDD patients and reported that the neocortical form was observed in 47% of PD patients and 67% of PDD patients, with no significant difference [261]. A large retrospective clinicopathological study of 106 autopsy patients revealed that only 20% of patients with cortical Lewy-related pathology were clinically diagnosed with PDD or DLB [262]. In addition to cortical pathology, a recent study compared the severity of Lewy-related pathology in the midbrain and reported that the amount of Lewy-related pathology in the substantia nigra and midbrain tectum/tegmentum was not different among PD, PDD, and DLB [67].

In summary, while several studies indicate a higher severity of cortical Lewy-related pathology in PDD and DLB compared to PD, other reports suggest that cortical Lewy-related pathology alone may not be a definitive marker for dementia. These findings highlight the complexity of the relationship between Lewy-related pathology and dementia. Lewy bodies in the substantia nigra were observed in a constant proportion of neurons in PD and DLB regardless of symptoms or disease duration, suggesting that Lewy bodies are removed at the death of neurons harboring them [263]. This phenomenon may complicate the correlation between Lewy-related pathology and clinical manifestations. Furthermore, comorbid conditions including amyloid and tau (discussed in detail below) may contribute to cognitive impairment.

Regarding the severity of cognitive impairment and Lewy-related pathology, a clinicopathological study with 44 PDD patients demonstrated that severity of cognitive impairment (Reisberg’s global deterioration scale) correlated with the number of cortical Lewy bodies [264]. Furthermore, the same authors showed that the number of Lewy bodies in the cingulate gyrus, frontal gyrus, temporal gyrus, amygdala, straight gyrus, and angular gyrus correlated with the severity of cognitive impairment [265]. Another study with 88 consecutive autopsies of PD or PDD (9 without cognitive impairment and 79 with cognitive impairment) revealed that the Mini-Mental State Examination score correlated with the distribution of Lewy-related pathology (i.e. Braak PD stage) [266]. Additionally, it was also reported that the severity of Lewy-related pathology in PD correlated with the final score on the Mini-Mental State Examination [267] and the rate of its decline [268]. Using another dementia scale, one study demonstrated a correlation between the density of Lewy-related pathology in the cortex and the nucleus basalis of Meynert and the clinical dementia rating [81].

These studies suggest that the degree of Lewy-related pathology may affect the severity of cognitive impairment in Lewy body disease; however, the brain regions that contribute to this relationship vary between studies. These differences may be attributed to the retrospective nature of many of the studies with varying degrees of accuracy of clinical information and the limited number of patients involved. Future clinicopathologic studies with a large number of prospectively followed-up patients are warranted.

### Visual hallucinations

There are also studies that have explored clinicopathological correlations for symptoms other than cognitive impairment. Core clinical features of DLB include recurrent visual hallucinations, cognitive fluctuations, RBD, and parkinsonism [1]. Recurrent complex visual hallucinations are frequently observed in patients with Lewy body dementia and the prevalence of visual hallucinations in DLB is about 60% [46, 52]. Patients with PDD and DLB experience well-formed hallucinations including people or animals [46]. The underlying pathology causing visual hallucinations is thought to be Lewy-related pathology rather than tau [269–272]. A study with 129 autopsies of PD (69 with dementia) examined the severity of cortical Lewy pathologies and revealed that patients with visual hallucinations had a greater cortical Lewy pathology severity [256]. Another pathological study examined the correlation between visual hallucinations and Lewy body burden in individual brain regions and revealed that patients with visual hallucinations exhibited a greater number of Lewy bodies in the parahippocampal cortex and amygdala [273]. Interestingly, when patients were divided by the time of onset of visual hallucinations, those with early-onset visual hallucinations had more Lewy bodies in the parahippocampal gyrus and temporal lobe, whereas those with late-onset of visual hallucinations showed no difference from those without visual hallucinations. This suggests a time lag between the onset of visual hallucinations and the formation of Lewy-related pathology. Another study investigated Lewy-related pathology in 19 autopsied brains of patients with DLB, 14 of whom had visual hallucinations [274]. Although the secondary visual pathway and amygdala showed more severe Lewy-related pathology than the primary visual pathway, no significant differences in pathological findings were observed between patients with and without visual hallucinations. These studies highlight the complex relationship between visual hallucinations and the distribution of Lewy-related pathology in DLB and warrant future research.

### Cognitive fluctuations

Fluctuations in DLB include waxing and waning of cognition, functional abilities, and arousal, and such fluctuations were observed in 63% of DLB patients [275]. A clinicopathological study demonstrated that patients with a distribution of Lewy bodies in the neocortex were more prone to fluctuations in their cognition, although the fluctuations were assessed using variation across annual average cognitive decline instead of hourly- or daily-based evaluations [82]. Additionally, functional imaging studies have suggested that the thalamus [276–278], basal ganglia [277, 279, 280], parietal lobe [279, 281, 282], occipital lobe [276, 281], or frontoparietal connections [283, 284] are potentially responsible for fluctuations in cognitive status [285, 286]. These fluctuations, however, are dynamic phenomena of very short duration, and pathological validation of the responsible regions remains elusive.

### REM sleep behavior disorder

During REM sleep, the normal muscle state is characterized by atonia, which is an almost complete paralysis of the skeletal muscles. RBD is a parasomnia with dream-enactment behaviors caused by a loss of REM sleep muscle atonia [287, 288]. The involvement of the brainstem nuclei in the pathogenesis of RBD has been suggested [289–291]. Patients with α-synucleinopathy often present with RBD long before the onset of motor symptoms or cognitive decline [1, 292–294]. Regarding the distribution of α-synuclein pathology, a study demonstrated that PD patients with RBD exhibited more severe α-synuclein burden in many brain regions, including the brainstem and cortices, compared to those without RBD [295]. Additionally, PD patients with RBD had a higher positive rate of phosphorylated α-synuclein in colon biopsies compared to those without RBD, suggesting that PD patients with RBD have a more widespread distribution of α-synuclein pathology [296]. Attempts have been made to identify the responsible foci of RBD by pathology, however, no convincing results have been obtained at present. A neuropathological study showed that cholinergic neuron counts in the pedunculopontine and laterodorsal tegmental nuclei were comparable between DLB and MSA patients with and without RBD [297]. Another study examined neuronal loss and α-synuclein burden in the pedunculopontine/laterodorsal tegmental nucleus and locus coeruleus between Lewy body disease patients with and without RBD, which found no difference [298]. Recently, in addition to cholinergic neurons [299, 300], the involvement of GABAergic and glutamatergic neurons has been suggested as a possible pathogenesis of RBD [301–305]. While Lewy-related pathology has been observed in GABAergic and glutamatergic neurons [306–308], the correlation between these findings and RBD requires further investigation. Additionally, studies have suggested the involvement of orexin neurons in RBD [309, 310]; however, orexin neurons are generally considered to lack Lewy-related pathology despite its presence in the hypothalamus [311]. Therefore, further research is needed to determine whether RBD and orexin neurons are indeed related in α-synucleinopathy.

### Other clinical manifestations

Clinicopathological studies have also been conducted on other manifestations of Lewy body dementia including daytime sleepiness and psychiatric symptoms. In a study examining sleep patterns in 61 DLB and 26 AD patients, DLB patients frequently demonstrated shorter initial sleep latency on multiple sleep latency tests and higher Epworth Sleepiness Scale scores [312]. Furthermore, among 20 DLB patients who underwent autopsy, 12 were classified as diffuse Lewy body disease and 8 as transitional Lewy body disease, suggesting that excessive daytime sleepiness can occur even in the absence of cortical Lewy bodies. Conversely, an analysis of 211 autopsy cases from the Honolulu-Asia Aging Study revealed that while 17.5% (20/114) of patients without Lewy-related pathology exhibited excessive daytime sleepiness, this symptom was present in 43.9% (25/57) of patients with neocortical Lewy-related pathology (Braak PD stage ≥ 5), demonstrating a correlation between the distribution of Lewy-related pathology and daytime hypersomnolence [313].

Regarding psychiatric symptoms, several studies have demonstrated associations between the distribution of Lewy-related pathology and clinical manifestations. In a study of 112 DLB patients, persistent delusions were found to increase with more widespread Lewy body distribution, occurring in 11% of brainstem-predominant, 32% of transitional, and 37% of diffuse Lewy body disease cases [270]. Another study revealed a greater number of Lewy bodies in the dorsal raphe nucleus in Lewy body disease with delusion compared to those without delusion [314]. A recent study using National Alzheimer’s Coordinating Center Registry data, which included 658 cases of Lewy body disease, demonstrated an increased frequency of delusions, anxiety, depression, and apathy as Lewy-related pathology spread from the brainstem to limbic structure and neocortex [315].

Additionally, a small number of clinicopathologic studies has been performed on atypical symptoms, including aphasia and behavioral changes. A large brain bank study identified 11 diffuse Lewy body disease patients presenting with corticobasal syndrome among 523 autopsy-confirmed diffuse Lewy body disease patients, and showed that these patients had more Lewy bodies in the motor cortex than those with clinically probable DLB [316]. Patients with diffuse Lewy body disease who clinically presented with logopenic progressive aphasia had a greater number of Lewy bodies in the middle frontal cortex, inferior parietal cortex, and anterior cingulate gyrus compared to those diffuse Lewy body disease patients clinically presenting with DLB [317]. These studies suggest that not only the type of accumulated protein but also where that protein accumulates may influence clinical presentation.

## Propagation hypothesis of pathological α-synuclein

Braak et al. conducted immunostaining for α-synuclein in autopsy cases of PD and clinically asymptomatic “incidental” Lewy body disease to reveal a gradient of pathological “spread” from the brainstem to the cerebrum. Based on this observation, they hypothesized that Lewy-related pathology propagates through the nervous system [181, 318, 319]. This study established a paradigm for subsequent research by translating the spatial distribution of lesions into a temporal axis. This propagation hypothesis was supported by several reports demonstrating that α-synuclein accumulation was observed within fetal nigral tissue transplanted into PD patients [320–322]. Additionally, experimental replication of α-synuclein propagation in the brain was achieved by inoculating synthetic α-synuclein preformed fibrils or seeds extracted from the brains of PD patients into the basal ganglia of mice [323, 324] and non-human primates [325, 326]. In recent years, studies in animals have reported the propagation of Lewy-related pathology to the cerebrum and brainstem after injection of preformed fibrils or seeds into the gastrointestinal tract [327, 328] or olfactory bulb [329–332].

In most cases of Lewy body disease without comorbid pathologies, Lewy-related pathology follows a stereotypical ascending pattern from the brainstem to the cerebrum, as confirmed by a number of studies replicating Braak’s hypothesis [333]. However, in Lewy body disease with comorbid AD, Lewy-related pathology in the amygdala is found from an early stage and even in the absence of Lewy-related pathology in the brainstem [192, 193, 334]. This underscores the heterogeneity of the propagation process.

### Origin of pathological α-synuclein

Given that pathological α-synuclein propagates and spreads throughout the brain, then where does the pathological α-synuclein originate? Braak et al. proposed that the gastrointestinal tract as the origin of pathological α-synuclein [319]. This proposal was based on their observation that the dorsal motor nucleus of the vagus nerve and/or the olfactory bulb are the sites where Lewy-related pathology is detected in the least affected brain, as well as on the findings that Lewy bodies are observed in the enteric nervous system [335, 336]. Since the vagus nerve, which innervates the gut, retrogrades to enter the dorsal motor nucleus of the vagus nerve in the medulla, it was hypothesized that the pathological α-synuclein originating in the gut could enter the central nervous system via the vagus nerve. Epidemiological data indicating a lower risk of developing PD in individuals who have undergone vagotomy [337, 338] along with the detection of Lewy bodies in intestinal specimens collected before the onset of motor symptoms [204, 339–342], are considered circumstantial evidence supporting the gastrointestinal origin of Lewy-related pathology. In contrast, there have been no cases where Lewy-related pathology was detected exclusively in the gastrointestinal tract [343–345], whereas several cases have been reported in which Lewy-related pathology was observed only in the olfactory bulb [183, 191, 346], cardiac sympathetic nerves [183, 347], or adrenal glands [213]. These cases suggest that the origin of Lewy-related pathology may be other than the gastrointestinal tract. Other hypotheses have been proposed, including the olfactory bulb as the origin [191], both the gastrointestinal tract and the olfactory bulb as origins [348–355], or a multicentric origin [356–361]. Each of these hypotheses lacks conclusive evidence or is refuted by contrary evidence.

### Prion-like propagation mechanisms

Experimental evidence from both in vitro and in vivo models demonstrates prion-like propagation of α-synuclein [242, 362–365]. Luk et al. demonstrated that introducing exogenous recombinant α-synuclein fibrils into cells overexpressing α-synuclein induces the recruitment of endogenous α-synuclein and the formation of Lewy body-like inclusions [366]. Furthermore, extracellular α-synuclein oligomers can cross cell membranes and trigger intracellular aggregation of endogenous α-synuclein in both primary neurons and neuronal cell lines [367]. Aulić et al. showed that a single exposure of SH-SY5Y cells to recombinant α-synuclein amyloid fibrils could induce aggregation of endogenous wild-type α-synuclein, even without overexpression and that the seeded aggregates persisted and propagated over several cell passages [368]. In vivo models also provide evidence for α-synuclein prion-like propagation. Desplats et al. demonstrated direct α-synuclein transmission by showing inclusion body formation in mouse cortical neural stem cells stereotactically injected into the hippocampus of transgenic mice that overexpress human α-synuclein [369]. In another study, intracerebral injection of brain homogenates from symptomatic α-synuclein transgenic mice or recombinant α-synuclein fibrils into young, asymptomatic α-synuclein transgenic mice rapidly induced widespread Lewy-related pathology-like inclusions, leading to motor symptoms and reduced survival [370]. Furthermore, stereotactic injection of insoluble α-synuclein from brains of patients with DLB and recombinant α-synuclein fibrils into wild-type mouse brains resulted in abnormal phosphorylation of endogenous mouse α-synuclein and its time- and region-dependent propagation from the injection site [324].

### Disease-specific propagation patterns

Notably, multiple studies using patient brain homogenates have demonstrated distinct α-synuclein propagation properties among synucleinopathies, including PD, DLB, and MSA. In vitro studies revealed fundamental differences in seeding properties: MSA-derived α-synuclein exhibits diffuse inclusion-forming capacity in both soluble and insoluble fractions with more efficient seeding activity, whereas PD-derived α-synuclein shows localized inclusion-forming capacity only in the insoluble fraction [371]. Cell-based assays using α-synuclein mutant HEK cells demonstrated distinct aggregation patterns, with MSA inducing large aggregates in most cell lines except for E46K, DLB forming small aggregates in all cell lines, and PD producing very small aggregates [372]. In vivo propagation experiments have consistently demonstrated the greater pathogenic potential of MSA-derived α-synuclein across different mouse models. Studies using α-synuclein A53T transgenic mice showed that MSA brain homogenate induced severe motor impairment and widespread α-synuclein pathology, while DLB brain homogenate produced no neurological symptoms and only localized pathological changes [373, 374]. Similarly, experiments in wild-type mice demonstrated that MSA brain homogenate induced more severe and widespread α-synuclein pathology with stronger microglial activation compared to PD brain homogenate [375]. Early studies confirmed that MSA brain tissue induced neurodegeneration in both cell and transgenic mouse models, while PD brain tissue did not [376]. Another study using wild-type rats further validated these findings, showing that MSA-derived α-synuclein assemblies are more aggressive and potent in inducing neuropathological and behavioral phenotypes compared to PD and DLB assemblies [377]. These findings collectively support the “α-synuclein strain hypothesis” that proposes disease-specific prion-like propagation mechanisms among synucleinopathies.

### Computational approaches to propagation analysis

Recent advances in computational pathology have provided new insights into α-synuclein propagation patterns. Pathological data obtained from postmortem brains represent single time points at death for each patient, making it challenging to track progression of pathology through brain regions over time within the same patient. To address this limitation, the Subtype and Stage Inference (SuStaIn) algorithm enables probabilistic reconstruction of temporal disease progression patterns [378, 379] from various types of cross-sectional data such as neuroimaging [380–389] and neuropathology [390–393]. Several studies have explored pathological progression patterns by applying the SuStaIn algorithm to Lewy-related pathology data. Mastenbroek et al. utilized semi-quantitative scores across ten brain regions in 814 autopsy cases, identifying three distinct progression patterns [391]. In 82% of cases, Lewy-related pathology initially accumulated in the olfactory bulb, with subsequent involvement of either the limbic system (61% of total cases) or brainstem (21% of total cases). The remaining 18% exhibited initial pathology in the brainstem. Another study by Andersen et al. applied the algorithm to two datasets (173 and 129 cases) [393]. With notable inclusion of many cases with incidental Lewy body disease and assessment of peripheral tissue pathology, their analysis identified brain-first (51%) and body-first (49%) subtypes, with the latter subdivided into parasympathetic and sympathetic variants. These approaches may enable the extraction of temporal data through pseudo-time analysis from static, single-timepoint pathological data, potentially revealing disease progression patterns.

Although the hypothesis of pathological α-synuclein propagation has been widely studied and supported by experimental evidence, the origin of pathological α-synuclein remains to be elucidated. Advancing our understanding of the origins and pathways of α-synuclein propagation will be crucial in developing effective interventions for Lewy body dementia.

## α-synuclein oligomer and its clinical implication

### Toxic mechanisms of α-synuclein oligomers

**Fig. 4:**
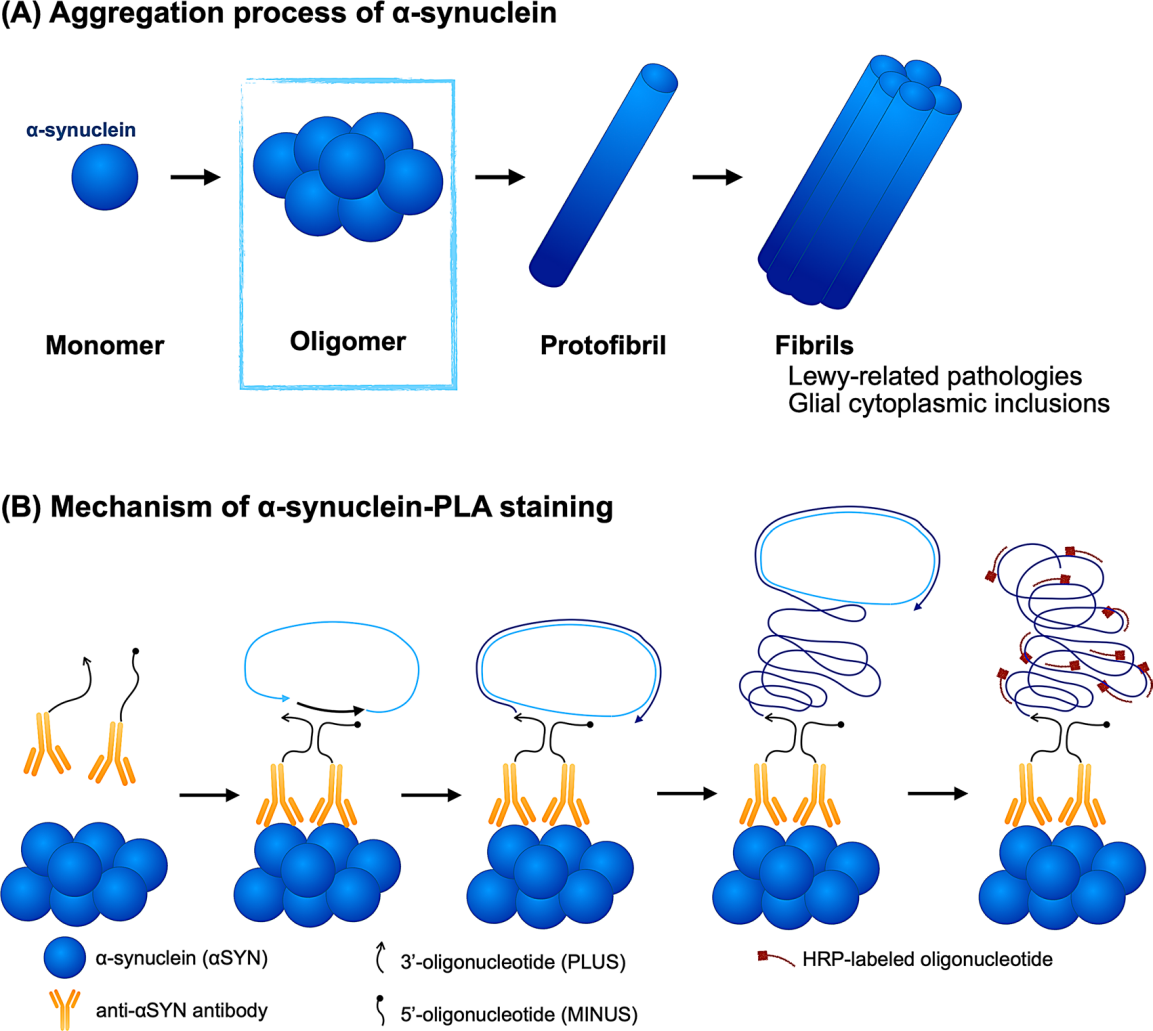
α-synuclein oligomers. (**A**) The aggregation process of α-synuclein. Multiple molecules form an early-stage aggregate of α-synuclein, known as an α-synuclein oligomer. (**B**) an anti-α-synuclein antibody conjugated with one oligonucleotide (PLUS) and an anti-α-synuclein antibody conjugated with another oligonucleotide (MINUS) are applied to the tissue. These antibodies bind in close proximity only in the presence of α-synuclein oligomers. This proximity allows subsequent reactions (ligation and amplification) to occur, enabling the visualization of α-synuclein oligomers

In the protein aggregation process, the intermediates from monomers to fibrils are termed oligomers, which are transient, heterogeneous higher-order protein aggregates [394] (Fig. 4A). In α-synucleinopathies, while Lewy-related pathology is the late-stage aggregates of α-synuclein, early-stage α-synuclein aggregates, known as α-synuclein oligomers, are attracting more attention in the pathogenesis. Mounting evidence has demonstrated that such α-synuclein oligomers are more toxic than Lewy-related pathology [395, 396]. Various mechanisms have been proposed for the toxicity of oligomeric species of synuclein based on cellular and animal studies. Among these toxicity pathways, membrane disruption is considered the primary [395, 397–406]. A recent study demonstrated that α-synuclein oligomers had a higher affinity for the membrane compared to monomeric α-synuclein and that they replace monomers on the membrane [406]. Other targets include mitochondrial dysfunction [407–412], synaptic impairment [401, 407, 413–419], endoplasmic reticulum stress [420–423], and oxidative stress [396, 424]. Additionally, several studies have suggested that α-synuclein oligomers elicit an inflammatory response, resulting in neuronal damage [424–428].

### Detection methods for α-synuclein oligomers

Methods for detecting α-synuclein oligomers include enzyme-linked immunosorbent assays [429–431], Förster resonance energy transfer-based techniques [432], and nanopore-based single-molecule detection [433]. These approaches can detect α-synuclein oligomers with high sensitivity in samples such as biological fluids, including cerebrospinal fluid, blood, and brain homogenates, as well as cell lysates. However, none of these methods preserve or provide information on the topographical distribution of α-synuclein oligomers within brain regions. Immunohistochemistry using oligomer-specific antibodies has been attempted to visualize α-synuclein oligomers in pathological sections; however, a systematic validation study has shown that widely used conformation-specific or oligomer-specific monoclonal antibodies bind not only to α-synuclein oligomers but also to fibrils and monomers [434]. This cross-reactivity limits the ability of immunohistochemistry to distinguish between oligomers and other aggregated forms of α-synuclein in tissue. To address this issue, a recent technique has enabled visualization of α-synuclein oligomers on histopathological slides. Proximity Ligation Assay (PLA) was originally developed as a method to detect endogenous protein interactions [435]. Fig. 4B illustrates how this method detects α-synuclein oligomers. By combining antigen-antibody reactions with in situ DNA amplification techniques, target proteins can be detected with high sensitivity and specificity. Two types of oligonucleotides are conjugated to antibodies against two target proteins, and only when the target proteins are in close proximity (within 40 nm), the two oligonucleotides are ligated, creating a template for amplification. This template is then amplified by rolling circle amplification. Complementary oligonucleotides labeled with horseradish peroxidase hybridize to the amplified product, and the signal is visualized by adding a substrate. This method was applied to detect α-synuclein oligomers by binding two different oligonucleotides to the same α-synuclein antibody [22, 428, 436–440]. This α-synuclein-PLA staining detected α-synuclein oligomers in morphologically normal neurons. Such α-synuclein oligomers were found in brain regions that were mildly affected [436]. Moreover, two independent research groups have successively reported that α-synuclein oligomers are distributed even in patients with *LRRK2*-associated PD who did not have Lewy-related pathology [441, 442]. The application of α-synuclein-PLA staining may reveal early α-synuclein pathology that has not been previously visualized. Some studies suggest that endogenous α-synuclein exists as helically folded tetramers [443, 444], while other research indicates it exists as disordered monomers [445]. Previous experiments using α-synuclein PLA staining did not observe many signals in control subjects [22, 436, 439, 441, 442, 446, 447]. Theoretically, α-synuclein PLA staining visualizes α-synuclein oligomers by recognizing two or more α-synuclein molecules in close proximity, typically within 40 nm. Physiological tetramers are tightly folded structures resistant to aggregation and may not efficiently expose the epitopes or spatial configurations necessary for PLA probe binding and ligation. Consequently, tetrameric structures might not be effectively detected by α-synuclein-PLA. Furthermore, physiological tetramers are fragile during tissue processing, and their detection requires preservation of lipid-protein interactions, which are lost during standard histological procedures. In contrast, pathological oligomers are more stable, likely surviving such processing and being detectable by PLA.

### Clinical implications and biomarker potential

Biochemically, α-synuclein oligomers have been detected in human postmortem brain extracts from patients with PD and DLB [243]. Significantly more synuclein oligomers were found in PD brains than in AD brains without Lewy-related pathology [429]. Furthermore, α-synuclein oligomers have also been detected in cerebrospinal fluid in PD and DLB, indicating their potential use as a biomarker for the underlying α-synuclein pathology [430, 448–452]. Hansson et al. demonstrated that α-synuclein oligomer levels in cerebrospinal fluid were higher in PDD compared to normal controls and AD; in DLB they were not significantly different from those in controls but higher than in AD [453]. van Steenoven et al. demonstrated that a combination of tau and α-synuclein oligomers in cerebrospinal fluid could differentiate DLB and AD [454].

Regarding the clinical implication of α-synuclein oligomers, Majbour et al. revealed that patients with advanced disease (Hoehn & Yahr stage II or higher) had significantly elevated α-synuclein oligomer levels in cerebrospinal fluid. With longitudinal assessment, they demonstrated that oligomer values at the 48-month timepoint strongly correlated with motor symptom severity [451]. Furthermore, a recent study demonstrated that PDD had more abundant α-synuclein oligomers in the hippocampus compared to PD without cognitive impairment, while Lewy-related pathology was comparable between those with and without cognitive impairment [447]. An association between cognitive impairment and α-synuclein oligomers has also been suggested in MSA [440, 446]. Additionally, another recent study in DLB found that patients with rapid cognitive decline had more abundant α-synuclein oligomers in the CA1 subfield of the hippocampus compared to those with slow cognitive decline [455]. With this recently developed visualization technique, further studies on the clinicopathological correlates of α-synuclein oligomers are warranted.

## Comorbid pathology – frequency and clinical impact

**Fig. 5:**
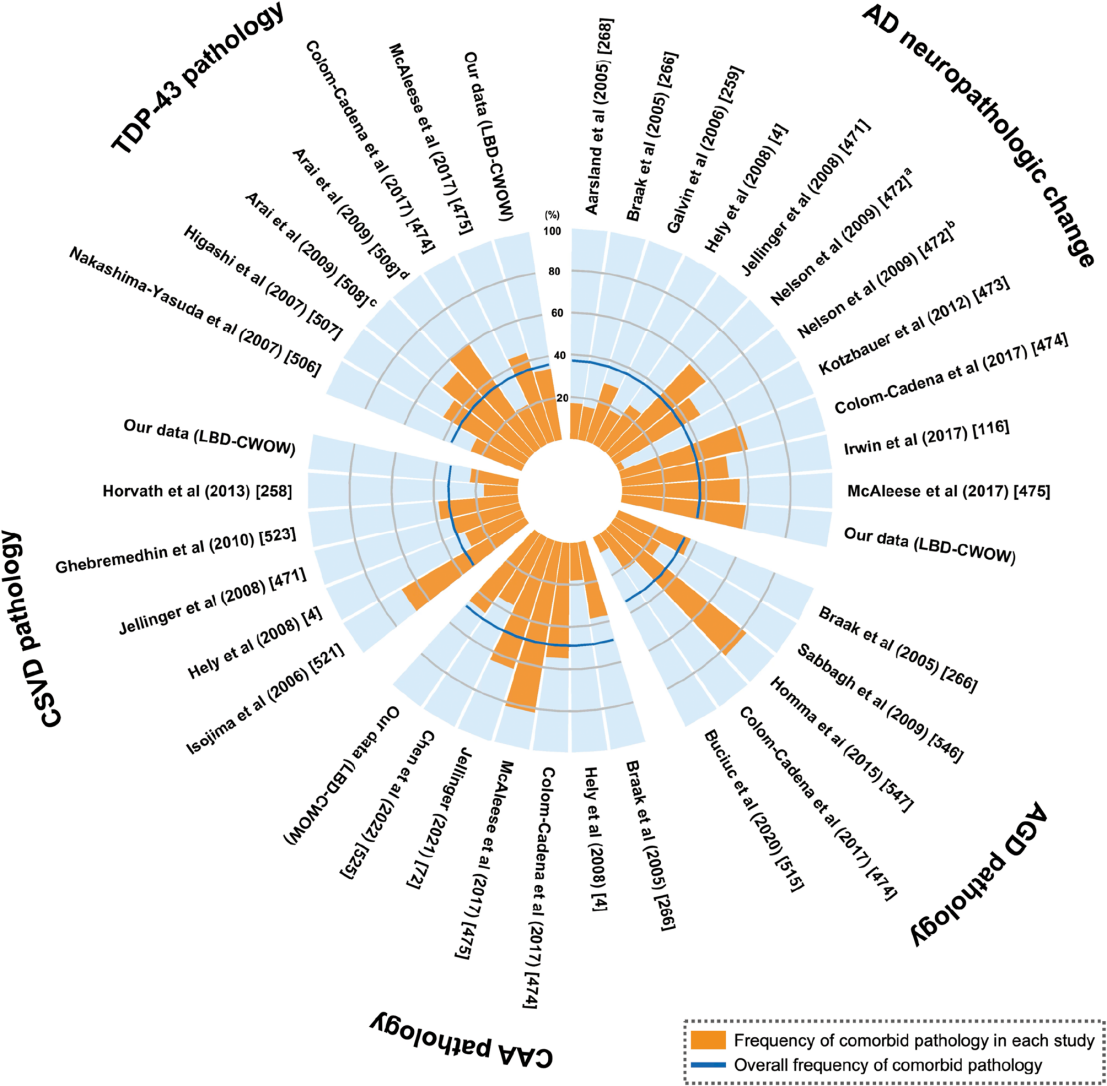
Frequency of each comorbid pathology. AD, Alzheimer’s disease; AGD, argyrophilic grain disease; CAA, cerebral amyloid angiopathy; CSVD, cerebral small vessel disease; TDP-43, transactive response DNA binding protein 43 kDa; (**a**) data from University of Kentucky Alzheimer’s disease Center; (**b**) data from national Alzheimer’s coordinating center Registry; (**c**) data from Tokyo institute of Psychiatry; (**d**) data from Canadian collaborative cohort of related dementia study

Aging is the major risk factor for most neurodegenerative diseases [456] and cerebrovascular disease [457]. Therefore, comorbid pathologies have been reported in neurodegenerative disorders [458–463]. In this section, we summarize previous reports of comorbid pathology from various investigators and discuss the clinicopathological impact of each comorbid pathology. Figure 5 illustrates the frequencies of comorbid pathologies based on previous reports and data from our institution. The overall frequency of each comorbid pathology was estimated using a forest plot. When multiple reports were published from the same institution, the representative one was adopted.

### AD neuropathologic change

Among comorbid pathologies, AD neuropathologic change is the most commonly observed in Lewy body disease. The pathology of AD is characterized by a combination of neurofibrillary tangles (NFTs) and senile plaques. NFTs are composed of hyperphosphorylated tau, while senile plaques are made of amyloid β. The distribution of each pathology is assessed based on the Braak NFT stage [464] and Thal amyloid phase [465], respectively. Additionally, the density of neuritic plaques is assessed using the Consortium to Establish a Registry for Alzheimer’s Disease (CERAD) score [466]. AD neuropathologic change is taken into account to assess the pathologic findings in Lewy body disease [1] (Table 5). Population-based autopsy studies demonstrate that the prevalence of neuropathologically confirmed AD increases with age, with rates ranging from 12% to over 60% depending on age, cohort characteristics, and diagnostic criteria [83, 467–469].

**Table 5:** Association of pathology stage combinations with typical DLB syndrome likelihood

	Alzheimer disease neuropathologic change (NIA-AA research framework)		
Lewy-related pathology stage	none/low	intermediate	high
Diffuse neocortical	High	High	Intermediate
Limbic (transitional)	High	Intermediate	Low
Brainstem-predominant	Low	Low	Low
Amygdala-predominant	Low	Low	Low
Olfactory bulb only	Low	Low	Low

*DLB*, dementia with Lewy bodies; NIA-AA, National Institute on Aging and Alzheimer’s Association

With respect to the frequency of AD neuropathologic change in Lewy body dementia, in a community-based study conducted in the southern Rogaland County, Norway, 238 PD patients were identified and prospectively followed [268]. Of the 22 patients for whom autopsies were obtained, 18 had dementia, of which 15 had low, 3 had intermediate, and 0 had high probability of AD based on the National Institute on Aging (NIA)-Reagan Institute criteria [470]. Another study also reported that 13 out of 79 PDD patients had AD neuropathologic change [266]. Subsequent studies have reported varying frequencies of comorbid AD in PDD and DLB [4, 116, 259, 471–475]. In the Lewy Body Dementia Center Without Walls (CWOW), Mayo Clinic brain bank, comorbid AD pathology was observed in 215 patients out of 363 Lewy body dementia patients (59%). We employed the Mayo Clinic Florida NIA-Alzheimer’s Association (NIA-AA) operational criteria to define AD neuropathologic change as previously described [463, 476]. Table 6 summarizes the frequency of comorbid AD and definition of AD employed in each study. Using these data, a forest plot estimated the frequency of AD complications in Lewy body dementia at 37% (Fig. 5 and Supplementary Fig. S1).

**Table 6:** Frequency of comorbid Alzheimer’s disease pathology in Lewy body dementia

Author (year)	N of Lewy body dementia cases	N (%) of cases with AD	Facility/Region	Country	Definition of AD
Aarsland et al. (2005) [268]	18	3 (17%)	County of Rogaland	Norway	NIA-RI criteria [477]
Braak et al. (2005) [266]	79	13 (16%)	Medisch Spectrum Twente Hospital Group in Enschede	Netherlands	Braak staging [464]
Galvin et al. (2006) [259]	34	10 (29%)	Washington University Movement Disorder Center	USA	NIA-RI criteria [477]
Hely et al. (2008) [4]	17	3 (18%)	Multicenter in Sydney	Australia	NIA-RI criteria [477]
Jellinger et al. (2008) [471]	20	5 (25%)	Vienna Neurobiology brain bank	Austria	Braak staging [464]
Nelson et al. (2009) [472]	33	19 (58%)	University of Kentucky Alzheimer’s Disease Center	USA	NIA-RI criteria [477]
Nelson et al. (2009) [472]	374	175 (47%)	National Alzheimer’s Coordinating Center Registry data	USA	NIA-RI criteria [477]
Kotzbauer et al. (2012) [473]	32	1 (3%)	Movement Disorders Center at Washington University School of Medicine	USA	NIA-RI criteria [477]
Colom-Cadena et al. (2017) [474]	47	29 (62%)	Neurological Tissue Bank of the Biobanc-Hospital Clinic-IDIBAPS in Barcelona	Spain	NIA-AA guidelines [466]
Irwin et al. (2017) [116]	213	108 (51%)	Five academic institutions^†^	USA	NIA-AA guidelines [466]
McAleese et al. (2017) [475]	34	19 (56%)	Newcastle Brain Tissue Resource	UK	NIA-AA guidelines [466]
*Our data*	363	215 (59%)	Lewy Body Dementia Center Without Walls, Mayo Clinic brain bank	USA	Mayo Clinic Florida NIA-AA operational criteria [463, 476]

*AD*, Alzheimer’s disease; *NIA-RI*, National Institute on Aging–Reagan Institute; *NIA-AA*, National Institute on Aging and Alzheimer’s Association

^†^ Penn Udall Center for Excellence in Parkinson’s Disease Research (Philadelphia), Pacific Udall Center (Seattle and Portland), Alzheimer’s Disease Core Center (Philadelphia), Alzheimer’s Disease Research Center (Seattle), Layton Aging and Alzheimer’s Disease Center (Portland), Alzheimer’s Disease Research Center (Pittsburgh), and Sanders-Brown Center on Aging (Lexington)

To date, many studies have demonstrated direct molecular interactions of α-synuclein with amyloid β and tau. Co-incubation experiments have demonstrated that α-synuclein monomers and oligomers inhibit amyloid-β fibrillization while promoting toxic oligomer formation via the C-terminus of amyloid-β. [478, 479]. This suggests synergistic toxicity mechanisms between α-synuclein and amyloid-β. Another experiment shows that oligomeric forms of amyloid-β can trigger α-synuclein aggregation via heterogeneous primary nucleation [480]. Additionally, amyloid-β and α-synuclein have a cross-seeding effect in vitro with fibrils showing stronger seeding activity than oligomers [481]. Similarly, α-synuclein and tau exhibit bidirectional interactions. Direct binding between α-synuclein and tau has been demonstrated, with the C-terminus of α-synuclein selectively interacting with tau and promoting its aggregation [482–485]. Additionally, α-synuclein and tau have been shown to synergistically promote each other’s aggregation [482, 483, 486–490]. The C-terminal region of α-synuclein plays an important role and α-synuclein phosphorylation at Ser129 further enhances tau aggregation [483, 487]. These studies collectively provide experimental evidence for direct protein-protein interactions, cross-aggregation, and synergistic effects between α-synuclein and both amyloid-β and tau, supporting mechanistic links underlying their co-occurrence.

The clinicopathological impact of comorbid AD pathology has been studied. With respect to the impact on clinical diagnosis, when AD pathology is comorbid with Lewy body disease, the probability of accurate antemortem diagnosis decreases, and patients frequently receive a clinical diagnosis of AD alone [491]. Regarding the impact on clinical manifestations, in a study comparing PDD with and without AD, PDD with AD had a higher age at PD onset and at death, shorter time to onset of dementia, and shorter duration of PD symptoms before dementia onset [492]. Another study found more severe tau and amyloid pathology in addition to Lewy-related pathology in PDD compared to PD without dementia [493]. PD with and without AD pathology exhibited a different distribution of Lewy-related pathology and PD with AD pathology was more likely to develop dementia compared to PD without AD pathology [494].

In contrast, several studies have reported that AD comorbidity does not have a major impact on cognitive function. One comparative study of neuropsychological assessment reported that although visuospatial impairment was more severe in DLB with AD than DLB without AD, cognitive decline itself was not significantly different between DLB with or without AD [495]. In another study, while Boston Naming Test was worse in Lewy body dementia with AD, MMSE, Dementia Rating Scale, and category fluency were comparable in those with and without AD [496]. Furthermore, a recent study examined the effect of 11 pathological conditions, including AD and Lewy-related pathology, on cognitive decline and demonstrated that these pathologies could not fully explain the progression of cognitive decline [497]. Another recent study compared the rate of MMSE decline among pure AD, AD with diffuse Lewy body disease, and AD with mild Lewy body pathology (to the extent that diffuse distribution and density thresholds were not met) and found that AD with mild Lewy body pathology had the fastest MMSE decline rate [498]. Although it is difficult to explain why AD with mild Lewy body pathology has a faster MMSE decline than AD+diffuse Lewy body disease, the authors speculated that cortical Lewy bodies may have some protective mechanism against AD pathology. As has been reviewed, the impact of comorbid AD pathology on cognitive function in Lewy body dementia remains controversial and is not fully understood.

### TAR DNA-binding protein 43 (TDP-43) pathology

TAR DNA-binding protein 43 (TDP-43) is a ubiquitously expressed nuclear protein and plays an important role in essential cell functions, including regulation of RNA metabolism, RNA splicing, mRNA transport, microRNA maturation, translational regulation, and the formation of stress granules [499]. Pathological aggregates of TDP-43 have been identified in the cytoplasm of neurons and glial cells in brains of patients with amyotrophic lateral sclerosis and frontotemporal lobar degeneration [500, 501]. According to population-based autopsy studies, the prevalence of TDP-43 pathology in the general population ranges from 19% to 64% [502–505].

Comorbid TDP-43 pathology has been reported in neurodegenerative disorders including Lewy body disease and AD [460, 474, 475, 506–509]. Table 7 summarizes the frequency of comorbid TDP-43 pathology in Lewy body dementia in previous studies. In the Lewy Body Dementia CWOW, Mayo Clinic brain bank, comorbid TDP-43 pathology was observed in 119 out of 350 patients with Lewy body dementia (34%). Based on these data, the overall frequency of TDP-43 pathology in Lewy body dementia was 36% (Fig. 5 and Supplementary Fig. S2).

**Table 7:** Frequency of comorbid TDP-43 pathology in Lewy body dementia

Author (year)	N of Lewy body dementia cases	N (%) of cases with TDP-43	Facility/Region	Country	Definition of TDP-43
Nakashima-Yasuda et al. (2007) [506]	111	29 (26%)	Five academic institutions^†^	USA	Presence of TDP-43-positive inclusions on IHC with antibody against TDP-43 (rabbit, polyclonal, ProteinTech)
Higashi et al. (2007) [507]	11	5 (45%)	Juntendo University	Japan	Presence of TDP-43-positive inclusions on IHC with antibody against TDP-43 (rabbit, polyclonal, ProteinTech)
Arai et al. (2009) [508]	15	8 (53%)	Tokyo Institute of Psychiatry	Japan	Presence of TDP-43-positive inclusions on IHC with antibody against phosphorylated TDP-43 (pS409/410 and pS403/404) [510]
Arai et al. (2009) [508]	10	6 (60%)	Canadian Collaborative Cohort of Related Dementia study	Canada	Presence of TDP-43-positive inclusions on IHC with antibody against phosphorylated TDP-43 (pS409/410 and pS403/404) [510]
Colom-Cadena et al. (2017) [474]	47	10 (21%)	Neurological Tissue Bank of the Biobanc-Hospital Clinic-IDIBAPS in Barcelona	Spain	Presence of TDP-43-positive inclusions on IHC with antibody against TDP-43 (2E2–D3, mouse, monoclonal, Abnova)
McAleese et al. (2017) [475]	34	15 (44%)	Newcastle Brain Tissue Resource	UK	Presence of TDP-43-positive inclusions on IHC with antibody against phosphorylated TDP-43 (pS409/410–2, rabbit, polyclonal, Cosmo Bio)
*Our data*	350	119 (34%)	Lewy Body Dementia Center Without Walls, Mayo Clinic brain bank	USA	Presence of TDP-43-positive inclusions on IHC with antibody against phosphorylated TDP-43 (pS409/410, mouse monoclonal, Cosmo Bio) [511]

*TDP-43*, transactive response DNA binding protein 43 kDa

† Penn Udall Center for Excellence in Parkinson’s Disease Research (Philadelphia), Pacific Udall Center (Seattle and Portland), Alzheimer’s Disease Core Center (Philadelphia), Alzheimer’s Disease Research Center (Seattle), Layton Aging and Alzheimer’s Disease Center (Portland), Alzheimer’s Disease Research Center (Pittsburgh), and Sanders-Brown Center on Aging (Lexington)

Several studies have investigated molecular interactions between α-synuclein and TDP-43. One study demonstrated that the low complexity domain (amino acids 310–329) of TDP-43 binds to full-length α-synuclein, while the non-amyloid β component of α-synuclein binds to full-length TDP-43 [512]. Additionally, the prion-like pathogenic C-terminal domain of TDP-43 interacts with α-synuclein to generate hybrid fibrils [513, 514]. Interestingly, α-synuclein monomers, oligomers, and sonicated fibrils seeded TDP-43 prion-like pathogenic C-terminal domain monomers, whereas reverse seeding did not occur. This suggests that TDP-43 abnormal aggregation may be secondarily induced by α-synuclein aggregation.

Regarding the effect on clinical diagnosis, patients with hippocampal TDP-43 were less likely diagnosed with DLB during their life and often received an alternative diagnosis of AD [515]. For the impact on clinical manifestations, whereas TDP-43 pathology deteriorates cognitive function in AD [516], its impact in Lewy body dementia appears to be limited. One study revealed that while disease duration was not significantly different, age at death was higher in PDD with TDP-43 compared to PDD without TDP-43 [506]. Another study did not find significant differences in age at death, disease duration, NFT stage, and Lewy body stage between DLB with and without TDP-43 [507]. No significant differences were observed in age at onset of cognitive impairment, cognitive decline progression, and final MMSE score between DLB with and without TDP-43 pathology [475]. However, a recent study with larger number of patients demonstrated that Lewy body disease with limbic-predominant TDP-43 had lower MMSE and faster cognitive decline compared to those without TDP-43 [517]. Thus, TDP-43 may independently exacerbate cognitive function in Lewy body dementia, and further studies are warranted.

### Cerebral small vessel disease (CSVD)

CSVD encompasses a range of pathological changes affecting the small arteries, arterioles, capillaries, and venules in the brain and can cause dementia, i.e., vascular dementia [518–520]. Pathologically, CSVD is characterized by several features: cortical and subcortical infarction, hemorrhage, microinfarcts, microbleeds, white matter change, and cerebral amyloid angiopathy (CAA). Among them, CAA will be discussed separately in the next section. As the focus of this review is pathology, MRI studies are not discussed.

Population-based autopsy studies show CSVD prevalence of 19% to 39% in the general population [468, 469]. Frequency of comorbid CSVD in Lewy body dementia ranged from 16% to 68% (Table 8) [4, 258, 471, 521, 523]. In the Lewy Body Dementia CWOW, Mayo Clinic brain bank, comorbid CSVD was detected in 84 out of 363 patients with Lewy body dementia (23%). Based on these data, the overall frequency of CSVD in Lewy body dementia was 33% (Fig. 5 and Supplementary Fig. S3).

**Table 8:** Frequency of comorbid cerebral small vessel disease in Lewy body dementia

Author (year)	N of Lewy body dementia cases	N (%) of cases with CSVD	Facility/Region	Country	Definition of CSVD
Isojima et al. (2006) [521]	25	17 (68%)	Yokohama City University and Fukushimura Hospital	Japan	Presence of hemorrhage, infarcts, or microinfarcts
Hely et al. (2008) [4]	17	5 (29%)	Multicenter in Sydney	Australia	NA
Jellinger et al. (2008) [471]	66	22 (33%)	Vienna Neurobiology brain bank	Austria	Presence of lacunes, infarcts, microinfarcts, hemorrhage, white matter lesions, or subcortical arteriosclerotic encephalopathy [522]
Ghebremedhin et al. (2010) [523]	13	5 (38%)	Medisch Spectrum Twente Hospital Group in Enschede	Netherlands	Presence of arteriosclerotic, arteriolosclerotic, lipohyalinotic (fibrinoid necrosis), or microaneurysmal formation in small parenchymal brain vessels [524]
Horvath et al. (2013) [258]	109	17 (16%)	Geneva University Brain Bank	Switzerland	Presence of infarcts, microinfarcts, or lacunar state
*Our data*	363	84 (23%)	Lewy Body Dementia Center Without Walls, Mayo Clinic brain bank	USA	Presence of arteriolosclerosis with microinfarcts, microbleeds, or ischemic white matter changes [463]

*CSVD*, cerebral small vessel disease

In a study examining the correlation between the presence of CSVD and clinical symptoms in DLB, memory disturbance was more frequent in DLB with CSVD, and parkinsonism was more frequent in DLB with CSVD as initial manifestations, whereas there was no difference in the frequency of individual clinical symptoms over the entire clinical course [521]. In a recent study using National Alzheimer’s Coordinating Center Registry data, hemorrhage and microbleeds had weak positive correlation with digit span forward length, but not with memory or language domains, while infarcts, lacunar, and microinfarcts did not correlate with either battery [525]. Pathologically, an inverse association between Lewy body score and small vessel disease score has been reported, and the authors have proposed that CSVD may lower the threshold for symptom development [523]. Besides these findings, there are reports that CSVD is associated with parkinsonism [526] and autonomic dysfunction [527]; however, its impact on cognitive function in Lewy body dementia seems limited.

### Cerebral amyloid angiopathy (CAA)

CAA is pathologically defined by the amyloid β deposition in the walls of small and medium-sized blood vessels in the cerebral cortex and leptomeninges [528]. The prevalence of CAA in the general population ranges from 40% to 79% according to population-based autopsy findings [529–532]. In previous autopsy studies, the frequency of comorbid CAA ranged from 18% to 82% in Lewy body dementia (Table 9) [4, 72, 266, 474, 475, 525]. In the Lewy Body Dementia CWOW, Mayo Clinic brain bank, 131 out of 363 patients exhibited comorbid CAA (36%). The overall frequency of comorbid CAA was estimated at 49% (Fig. 5 and Supplementary Fig. S4). One study including 110 PDD and 60 DLB demonstrated that the frequency of comorbid CAA was higher in DLB than in PDD (90% vs. 50%) [72].

**Table 9:** Frequency of comorbid cerebral amyloid angiopathy in Lewy body dementia

Author (year)	N of Lewy body dementia cases	N (%) of cases with CAA	Facility/Region	Country	Definition of CAA
Braak et al. (2005) [266]	79	29 (37%)	Medisch Spectrum Twente Hospital Group in Enschede	Netherlands	Revesz et al. [533]
Hely et al. (2008) [4]	17	3 (18%)	Multicenter in Sydney	Australia	NA
Colom-Cadena et al. (2017) [474]	47	26 (55%)	Neurological Tissue Bank of the Biobanc-Hospital Clinic-IDIBAPS in Barcelona	Spain	Vonsattel et al. [534]
McAleese et al. (2017) [475]	34	28 (82%)	Newcastle Brain Tissue Resource	UK	Thal et al. [535]
Jellinger (2021) [72]	170	109 (64%)	Vienna Neurobiology brain bank	Austria	Love et al. [536]
Chen et al. (2022) [525]	363	131 (36%)	National Alzheimer’s Coordinating Center Registry data	USA	Vonsattel et al. [534]
*Our data*	360	164 (46%)	Lewy Body Dementia Center Without Walls, Mayo Clinic brain bank	USA	Presence of amyloid deposition on vessel walls by thioflavin-S fluorescent microscopy [463]

*CAA*, cerebral amyloid angiopathy

The severity of CAA has been associated with a rapid disease progression in DLB compared to PDD [72]. A recent study using National Alzheimer’s Coordinating Center Registry data demonstrated a negative correlation between comorbid CAA and logical memory in the intermediate likelihood DLB [525]. This correlation was not observed in the high likelihood DLB. A few studies have examined the clinical impact of comorbid CAA, and further research is needed.

### Argyrophilic grain disease (AGD)

AGD is a distinct tauopathy characterized by the presence of spindle- or comma-shaped tau aggregates (argyrophilic grains) [537, 538]. Clinically, AGD can present with cognitive impairment [539, 540]. Although the prevalence of AGD in the general population remains unclear from population-based autopsy studies, consecutive autopsy series report AGD prevalence rates of 5–9% [541–543]. In Lewy body dementia, the frequency of comorbid AGD ranged from 8% to 83% in autopsy studies (Table 10) [266, 474, 515, 546, 547]. In a study examining 367 autopsy cases, AGD was found in 18% of AD, 26% of PDD, 22% of DLB, and 29% of normal controls, with no significant differences in frequency among conditions. [546]. The overall frequency of comorbid AGD in Lewy body dementia is estimated at 34% (Fig. 5 and Supplementary Fig. S5). Whether comorbid AGD independently affects clinical manifestations, including cognitive decline and Lewy-related pathology, remains unstudied.

**Table 10:** Frequency of comorbid argyrophilic grain disease in Lewy body dementia

Author (year)	N of Lewy body dementia cases	N (%) of cases with AGD	Facility/Region	Country	Definition of AGD
Braak et al. (2005) [266]	79	29 (37%)	Medisch Spectrum Twente Hospital Group in Enschede	Netherlands	Braak et al. [544], Jellinger [537], and Iseki et al. [545]
Sabbagh et al. (2009) [546]	51	13 (25%)	Sun Health Research Institute Brain Donation Program	USA	Presence of grains on Gallyas silver staining
Homma et al. (2015) [547]	12	10 (83%)	Tokyo Metropolitan Neurological Hospital	Japan	Presence of argyrophilic grains on Gallyas–Braak staining and Saito staging [548]
Colom-Cadena et al. (2017) [474]	47	10 (21%)	Neurological Tissue Bank of the Biobanc-Hospital Clinic-IDIBAPS in Barcelona	Spain	Saito staging [548]
Buciuc et al. (2020) [515]	158	12 (8%)	Mayo Clinic Alzheimer’s Disease Research Center	USA	Presence of silver and tau-positive spindle-shaped lesions [537]

*AGD*, argyrophilic grain disease

## Conclusions

This review highlights the complex neuropathological landscape of Lewy body dementia, encompassing classical Lewy-related pathology, α-synuclein oligomers, and comorbid pathologies along with their clinical implications. While significant progress has been made in understanding the underlying pathology of the disease, many questions remain unanswered. Specifically, the origin of pathological α-synuclein, the detailed pathways of its propagation, and the clinical impact of α-synuclein oligomers and each comorbid pathology are still largely unresolved. Future research should focus on elucidating the origins of pathological α-synuclein, pathways of its propagation, as well as the interplay between α-synuclein and comorbid pathologies. Such efforts are essential to advancing our knowledge and improving clinical outcomes for individuals affected by Lewy body dementia.

## Electronic supplementary material

Below is the link to the electronic supplementary material.

Supplementary Material 1

## Data Availability

Not applicable.

## Abbreviations

AD: Alzheimer’s disease
AGD: Argyrophilic grain disease
CAA: Cerebral amyloid angiopathy
CERAD: Consortium to establish a registry for Alzheimer’s Disease
CSVD: Cerebral small vessel disease
CWOW: Center without walls
DLB: Dementia with Lewy bodies
DSM-5: Diagnostic and statistical manual of mental disorders, Fifth Edition
MMSE: Mini-Mental state examination
MSA: Multiple system atrophy
NFT: Neurofibrillary tangle
NIA: National institute on aging
NIA-AA: National institute on aging-Alzheimer’s association
PD: Parkinson’s disease
PDD: Parkinson’s disease dementia
PLA: Proximity ligation assay
RBD: Rapid eye movement sleep behavior disorder
SuStaIn: Subtype and stage inference
TDP-43: TAR DNA-binding protein 43
