# Supplementary material for: Neuropathology of Lewy body dementia: Lewy-related pathology, α-synuclein oligomers, and comorbid pathologies
Source: Mol Neurodegener. 2025 Nov 3;20:117. doi: 10.1186/s13024-025-00900-6 (PMC12581614; doi:10.1186/s13024-025-00900-6)
Supplement: Supplementary file 1 — Supplementary Material 1 [file 13024_2025_900_MOESM1_ESM.pdf]

Supplementary Materials for

Neuropathology of Lewy Body Dementia: Lewy-Related Pathology, α-Synuclein Oligomers, and Comorbid Pathologies

Hiroaki Sekiya, M.D., Ph.D.[1], Tomoyasu Matsubara, M.D., Ph.D.[2], Michael A. DeTure, Ph.D.[1], Dennis W. Dickson, M.D.[1]

1 Department of Neuroscience, Mayo Clinic, Jacksonville, Florida, USA

2 Department of Clinical Neuroscience and Therapeutics, Hiroshima University Graduate School of Biomedical and Health Sciences, Hiroshima, Japan

Corresponding authors:

Hiroaki Sekiya, MD, PhD, Email: sekiya.hiroaki@mayo.edu
Dennis W. Dickson, MD, Email: dickson.dennis@mayo.edu

This PDF file includes Supplementary Figures S1 to S5.

Figure S1. Frequency of comorbid AD pathology

Study	Number of AD pathology	LBD cases	Proportion with 95% CI	Weight (%)
Aarsland et al (2005) [268]	3	18	0.17 [0.00, 0.34]	7.77
Braak et al (2005) [266]	13	79	0.16 [0.08, 0.25]	8.88
Galvin et al (2006) [259]	10	34	0.29 [0.14, 0.45]	8.04
Hely et al (2008) [4]	3	17	0.18 [0.00, 0.36]	7.63
Jellinger et al (2008) [471]	5	20	0.25 [0.06, 0.44]	7.51
Nelson et al (2009)[a] [472]	19	33	0.58 [0.41, 0.74]	7.82
Nelson et al (2009)[b] [472]	175	374	0.47 [0.42, 0.52]	9.11
Kotzbauer et al (2012) [473]	1	32	0.03 [0.00, 0.09]	9.05
Colom-Cadena et al (2017) [474]	29	47	0.62 [0.48, 0.76]	8.23
Irwin et al (2017) [116]	108	213	0.51 [0.44, 0.57]	9.00
McAleese et al (2017) [475]	19	34	0.56 [0.39, 0.73]	7.84
Our data (LBD-CWOW)	215	363	0.59 [0.54, 0.64]	9.11
**Overall**			**0.37** [0.25, 0.49]	

Heterogeneity: $\tau^2 = 0.04$, $I^2 = 95.17\%$, $H^2 = 20.70$, $p < 0.001$

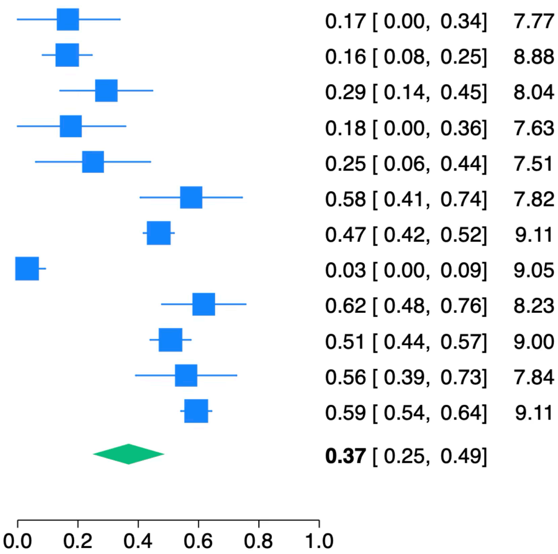

Random-effects REML model; *AD*, Alzheimer's disease; *LBD*, Lewy body dementia; [a] Data from University of Kentucky Alzheimer's Disease Center; [b] Data from National Alzheimer's Coordinating Center Registry.

Figure S2. Frequency of comorbid TDP-43 pathology

Study	Number of TDP-43 pathology	LBD cases	Proportion with 95% CI	Weight (%)
Nakashima-Yasuda et al (2007) [506]	29	111	0.26 [0.18, 0.34]	21.42
Higashi et al (2007) [507]	5	11	0.45 [0.16, 0.75]	7.05
Arai et al (2009)[a] [508]	8	15	0.53 [0.28, 0.79]	8.72
Arai et al (2009)[b] [508]	6	10	0.60 [0.30, 0.90]	6.73
Colom-Cadena et al (2017) [474]	10	47	0.21 [0.10, 0.33]	18.17
McAleese et al (2017) [475]	15	34	0.44 [0.27, 0.61]	13.91
Our data (LBD-CWOW)	119	350	0.34 [0.29, 0.39]	24.00
**Overall**			**0.36** [0.26, 0.45]	

Heterogeneity: $\tau^2 = 0.01$, $I^2 = 69.78\%$, $H^2 = 3.31$. $p = 0.02$

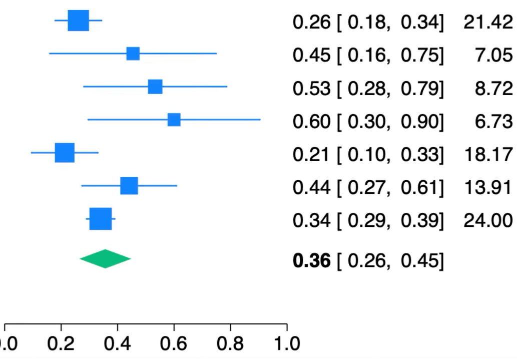

Random-effects REML model; *TDP-43*, transactive response DNA binding protein 43 kDa; *LBD*, Lewy body dementia; [a] Data from Tokyo Institute of Psychiatry; [b] Data from Canadian Collaborative Cohort of Related Dementia study.

Figure S3. Frequency of comorbid cerebral small vessel disease

Study	Number of CSVD pathology	LBD cases	Proportion with 95% CI	Weight (%)
Isojima et al (2006) [521]	17	25	0.68 [0.50, 0.86]	15.53
Hely et al (2008) [4]	5	17	0.29 [0.08, 0.51]	14.14
Jellinger et al (2008) [471]	22	66	0.33 [0.22, 0.45]	18.25
Ghebremedhin et al (2010) [523]	5	13	0.38 [0.12, 0.65]	12.27
Horvath et al (2013) [258]	17	109	0.16 [0.09, 0.22]	19.65
Our data (LBD-CWOW)	84	363	0.23 [0.19, 0.27]	20.16
**Overall**			**0.33** [0.19, 0.48]	

Heterogeneity: $\tau^2 = 0.03$, $I^2 = 91.41\%$, $H^2 = 11.64$, $p < 0.001$

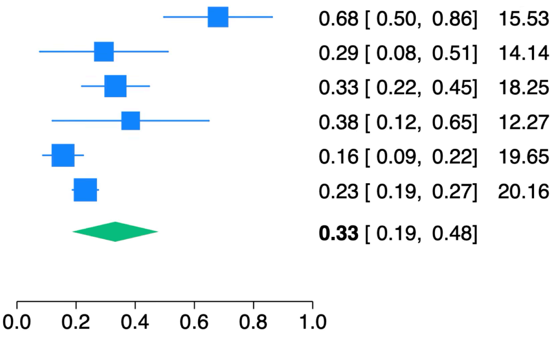

Random-effects REML model; *CSVD*, cerebral small vessel disease; *LBD*, Lewy body dementia

Figure S4. Frequency of cerebral amyloid angiopathy

Study	Number of CAA pathology	LBD cases	Proportion with 95% CI	Weight (%)
Braak et al (2005) [266]	29	79	0.37 [0.26, 0.47]	14.38
Hely et al (2008) [4]	3	17	0.18 [0.00, 0.36]	12.65
Colom-Cadena et al (2017) [474]	26	47	0.55 [0.41, 0.70]	13.61
McAleese et al (2017) [475]	28	34	0.82 [0.70, 0.95]	13.93
Jellinger (2021) [72]	109	170	0.64 [0.57, 0.71]	14.96
Chen et al (2022) [525]	131	363	0.36 [0.31, 0.41]	15.24
Our data (LBD-CWOW)	164	360	0.46 [0.40, 0.51]	15.22
**Overall**			**0.49** [0.34, 0.64]	

Heterogeneity: $\tau^2 = 0.04$, $I^2 = 95.68\%$, $H^2 = 23.13$, $p < 0.001$

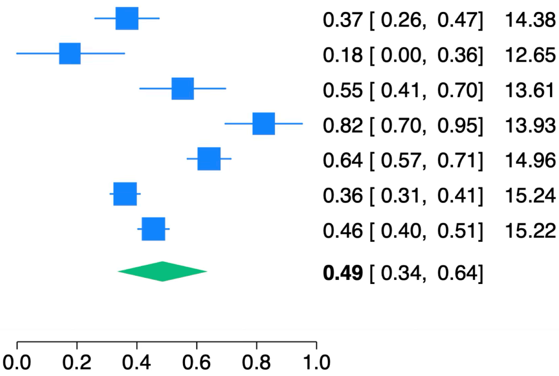

Random-effects REML model; *CAA*, cerebral amyloid angiopathy; *LBD*, Lewy body dementia

Figure S5. Frequency of argyrophilic grain disease

Study	Number of AGD pathology	LBD cases	Proportion with 95% CI	Weight (%)
Braak et al (2005) [266]	29	79	0.37 [0.26, 0.47]	20.36
Sabbagh et al (2009) [546]	13	51	0.25 [0.14, 0.37]	20.15
Homma et al (2015) [547]	10	12	0.83 [0.62, 1.00]	18.25
Colom-Cadena et al (2017) [474]	10	47	0.21 [0.10, 0.33]	20.19
Buciuc et al (2020) [515]	12	158	0.08 [0.03, 0.12]	21.06
**Overall**			**0.34** [0.10, 0.58]	

Heterogeneity: $\tau^2 = 0.07$, $I^2 = 96.58\%$, $H^2 = 29.21$, $p < 0.001$

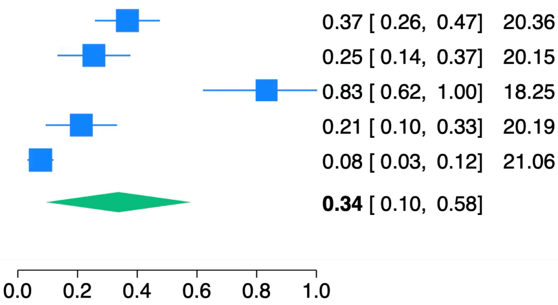

Random-effects REML model; *AGD*, argyrophilic grain disease; *LBD*, Lewy body dementia
